# Structural basis of human zinc-activated channel (ZAC) signaling and modulation

**DOI:** 10.1038/s41421-026-00878-5

**Published:** 2026-03-31

**Authors:** Zixuan Zhou, Yonghui Long, Yulin Chao, Chuanhui Yang, Yi-Quan Tang, Yilai Shu, Hongtao Zhu, Anders A. Jensen, Qianhui Qu

**Affiliations:** 1 Eye & ENT Hospital, Institutes of Biomedical Sciences, Shanghai Key Laboratory of Medical Epigenetics, International Co-laboratory of Medical Epigenetics and Metabolism (Ministry of Science and Technology), Department of Systems Biology for Medicine, Fudan University, Shanghai, China; 2 State Key Laboratory of Medical Neurobiology and MOE Frontiers Center for Brain Science, Institutes of Brain Science, Fudan University, Shanghai, China; 3 ENT Institute and Otorhinolaryngology Department of Eye & ENT Hospital, Fudan University, Shanghai, China; 4 Beijing National Laboratory for Condensed Matter Physics, Institute of Physics, Chinese Academy of Sciences, Beijing, China; 5 Department of Drug Design and Pharmacology, Faculty of Health and Medical Sciences, University of Copenhagen, Copenhagen, Denmark

**Keywords:** Cryoelectron microscopy, Ion channel signalling

## Abstract

Zinc (Zn^2+^) plays essential roles in a plethora of physiological processes, including key functions as a neuromodulator. The zinc-activated channel (ZAC) belongs to the Cys-loop receptor (CLR) superfamily of pentameric ligand-gated ion channels, which also comprises receptors for the important neurotransmitters acetylcholine, serotonin, GABA and glycine. In contrast to these classical CLRs, which have been extensively explored over decades, ZAC remains poorly characterized despite its potential significance in mammals. Here, we present several cryo-EM structures of human ZAC, including the ligand-free resting state, the Zn^2+^-bound state, and several antagonist-bound states. In the Zn^2+^-bound structure, Zn^2+^ ions bind to the subunit interfaces of the extracellular domain, corresponding to the canonical agonist-binding sites in the classical CLRs, and are primarily coordinated through cation‒π interactions with two aromatic residues. While the antagonist TTFB inhibits ZAC by insertion between the transmembrane M2 helices of adjacent subunits, *d*-tubocurarine acts in a dual manner by blocking the channel and interfering with agonist binding. Combined with mutagenesis and electrophysiological analysis, these evaluations highlight the distinctive structural and functional features of this atypical CLR.

## Introduction

Zinc (Zn^2+^) is the second most abundant transition metal ion in mammals and is a widely distributed trace element critical for numerous bodily functions, including cell growth and proliferation^1^. In addition to its structural and catalytic roles in more than 10% of human proteins, which play significant roles in the regulation of, for example, gene expression, the immune response, and antioxidant defenses, zinc also functions as a neuromodulator, with significant enrichment of free Zn^2+^ ions in specific brain regions such as the hippocampus, amygdala, and cerebral cortex^2^. Synaptic Zn^2+^ released at certain glutamatergic and GABAergic terminals plays important roles in maintaining proper neuronal circuitry function in the brain and spinal cord, in part by fine-tuning the activity of a range of ligand-gated and voltage-gated ion channels^3–6^. Both reduced and elevated zinc levels have been implicated in the development of various neuropathological conditions, including Parkinson’s disease, Alzheimer’s disease, epilepsy, depression, schizophrenia, attention deficit/hyperactivity disorder, and noise-induced hearing loss^7–11^.

Cys-loop receptors (CLRs) are pentameric ligand-gated ion channels that act as key mediators of fast synaptic transmission driven by the classical neurotransmitters acetylcholine (ACh), serotonin (5-HT), γ-aminobutyric acid (GABA) and glycine^12^. The nicotinic ACh, 5-HT_3_, GABA type A, and glycine receptors (nAChRs, 5-HT_3_Rs, GABA_A_Rs and GlyRs, respectively) play essential roles in the physiology and pathophysiology of nervous systems and serve as therapeutic targets for a variety of pathological conditions^13^.

The zinc-activated channel (ZAC) constitutes the sole member of the fifth branch of the mammalian CLR superfamily and exhibits low sequence homology (< 20% residue identity) with the other family members but most of the structural hallmark features of a CLR^4,14^. Orthologs of the human *ZACN* gene are present in the genomes of a wide range of mammalian species but are notably absent in the rat and mouse genomes^15,16^. ZAC appears to be abundantly expressed in the human body, as *ZACN* transcripts have been identified in several tissues, including the placenta, pancreas, liver, heart, stomach, and brain, where immunostaining has revealed ZAC protein expression in the hippocampus and dentate gyrus^15^. The expression of recombinant ZAC in mammalian cells or *Xenopus* oocytes results in the assembly of homomeric channels characterized by pronounced levels of spontaneous activity that are activated by Zn^2+^, Cu^2+^ and H^+^^17^. A recent study revealed that Zn^2+^ and H^+^ mediate ZAC activation primarily through interactions with the extracellular domain (ECD) of the receptor^13^. However, extensive mutational analyses targeting most of the conventional zinc-coordinating residues in proteins — histidine, cysteine, glutamate, and aspartate^18^ — in this domain failed to significantly impact Zn^2+^-induced ZAC signaling^13^, thus leaving the precise mechanism of Zn^2+^-evoked signal transduction through the receptor unresolved. ZAC exhibits nonselective permeability to the monovalent cations Na^+^, K^+^ and Cs^+^, whereas the receptor is inhibited by high (nonphysiological) concentrations of the divalent cations Ca^2+^ and Mg^2+^^15,17^. While the pharmacological toolbox available for ZAC is currently very limited, the CLR inhibitor *d*-tubocurarine (*d*-TC) and a series of *N*-(thiazol-2-yl)-benzamide analogs have been identified as relatively potent ZAC antagonists^15,19^.

During the preparation of this manuscript, cryo-EM structures of the ligand-free resting (apo) state of *ol*ZAC, a proposed ZAC ortholog from medaka fish (*Oryzias latipes*)^20^, and of the apo and Zn^2+^-bound states of the human ZAC^A152^ variant^21^ were published. In the present study, we determined several cryo-EM structures of human ZAC in different functional states. Comparison of the Zn^2+^-bound and apo states of ZAC reveals that Zn^2+^ ions occupy binding sites located at the five ECD subunit interfaces in the receptor, where its binding is formed mainly through (for Zn^2+^) atypical cation‒π interactions with a couple of aromatic residues. Analysis of the structures of wild-type (WT) ZAC and its high-frequency variant ZAC^A152^ establishes a plausible structural basis for the pronouncedly attenuated gating properties exhibited by the homopentameric variant receptor^22^. The selective ZAC antagonist *N*-(4-(tert-butyl)thiazol-2-yl)-3-fluorobenzamide (TTFB) inhibits the receptor by intercalating between the M2 transmembrane helices of adjacent protomers in the pentamer, a unique interference mode not previously observed in CLR/modulator structures. Finally, *d*-TC inhibits ZAC by binding sites both in the ECD and in the transmembrane channel pore. Collectively, these findings provide valuable and detailed insights into the distinct structural and functional characteristics of this atypical member of the CLR superfamily and into the molecular basis underlying its modulation by several small-molecule ligands.

## Results

### Overall architecture of human ZAC

**Fig. 1:**
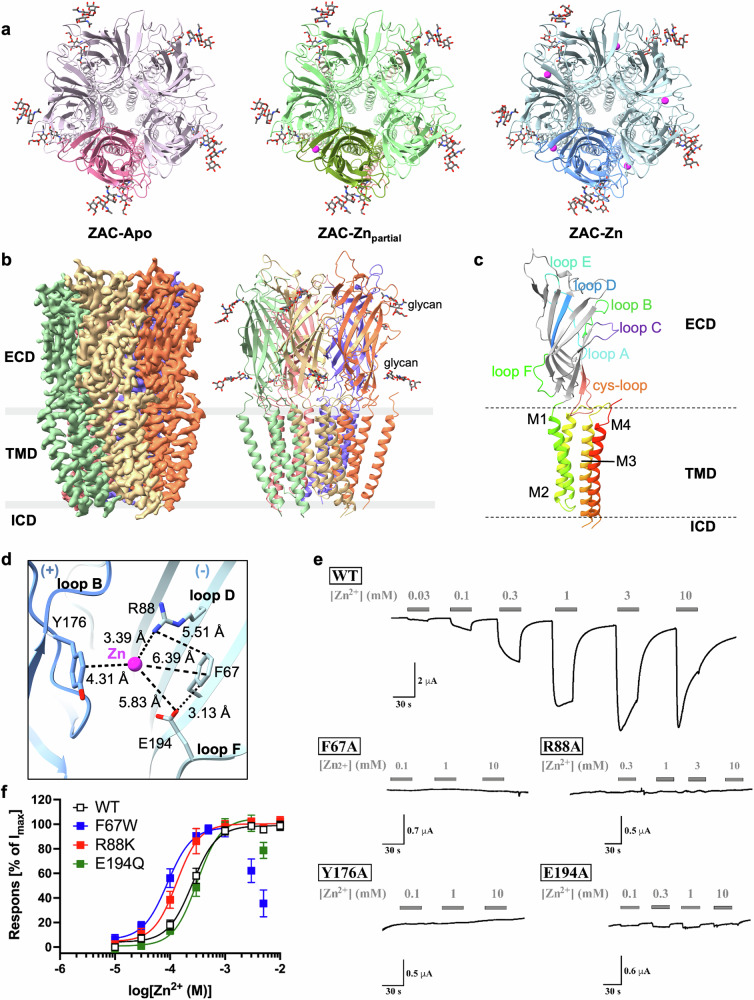
Cryo-EM structures of ZAC and its zinc-binding sites. **a** Top-down view comparing ZAC in three states: apo (left), one zinc ion bound (middle), and fully zinc bound (right). In each structure, a single subunit is distinctively colored for clarity: pale violet-red for ZAC-Apo, olive drab for ZAC-Zn_partial_, and cornflower blue for ZAC-Zn. Zinc ions are represented as magenta balls. **b** Cryo-EM density maps (left) and structural models of human ZAC in its apo state resolved at 3.03 Å, with each subunit colored uniquely. Extracellular *N*-glycosylations are depicted as gray sticks. **c** A detailed view of a single ZAC subunit. The N-terminal ECD is shown in gray, with loops A–F highlighted in different colors, and the TMD is depicted using a rainbow color scheme. **d** Detailed view of Zn^2+^–residue interactions in the zinc-binding site at the ECD subunit interface (indicated by dashed lines). The principal subunit interface (+) and the complementary subunit interface (−) are given in dark and light blue, respectively. **e** Representative traces of the current responses evoked by Zn^2+^ in *Xenopus* oocytes expressing WT ZAC and four ZAC mutants (F67A, R88A, Y176A, and E194A) in TEVC recordings. Representative traces of the current responses evoked by Zn^2+^ through the other zinc-binding site mutants are given in Supplementary Fig. S4. **f** Agonist concentration‒response relationships exhibited by Zn^2+^ in WT and mutant F67W, R88K and E194Q ZAC in *Xenopus* oocytes in TEVC recordings. Data are given as mean ± SEM normalized to the fitted I_max_ for each receptor and are based on the number of recordings for each receptor given in Supplementary Table S3. The fitted concentration‒response curves are based on all the data points in the graph, except for the data points for Zn^2+^ (3 and 5 mM) for F67W and for Zn^2+^ (5 mM) for E194Q. The average agonist properties of Zn^2+^ at WT ZAC and all zinc-binding site mutants are given in Supplementary Table S3.

Initial attempts to overexpress full-length human ZAC in HEK293 cells using transient transfection with a plasmid encoding ZAC with a C-terminally fused green fluorescence protein (GFP) and a Strep-II affinity tag yielded low amounts of protein of poor quality that was prone to aggregation. To obtain high-quality ZAC protein, we adopted the BacMam viral expression system and screened various detergents and buffer conditions for membrane solubilization and purification. The protein was solubilized in n-dodecyl-β-D-maltose (DDM) and cholesteryl hemisuccinate (CHS), purified with lauryl maltose neopentyl glycol (LMNG) and CHS, and subsequently reconstituted into nanodiscs with Brain Total Lipid. Cryo-EM visualization of the ZAC protein purified in the presence of 1 mM EDTA revealed a map at 3.35 Å resolution (ZAC-Apo), whereas incubation with 50 µM ZnSO_4_ yielded a final 2.85 Å map with only one obvious density in one of the five ECD subunit interfaces in ZAC, corresponding to a Zn^2+^ ion (ZAC-Zn_partial_). A Zn^2+^-fully bound (i.e., with Zn^2+^ ions bound to each of the five ECD subunit interfaces) map with a resolution of 2.62 Å (ZAC-Zn) was obtained by incubating the ZAC protein with 250 µM ZnSO_4_ prior to vitrification (Fig. 1; Supplementary Fig. S1 and Table S1). Notably, the ZAC protein strongly aggregated at higher ZnSO_4_ concentrations. These high-resolution density maps of ZAC allow accurate modeling of most regions, except the N-terminus (residues 1–46), the intracellular domain (ICD, residues 323–360), and the extracellular C-terminus (residues 396–412).

**Fig. 2:**
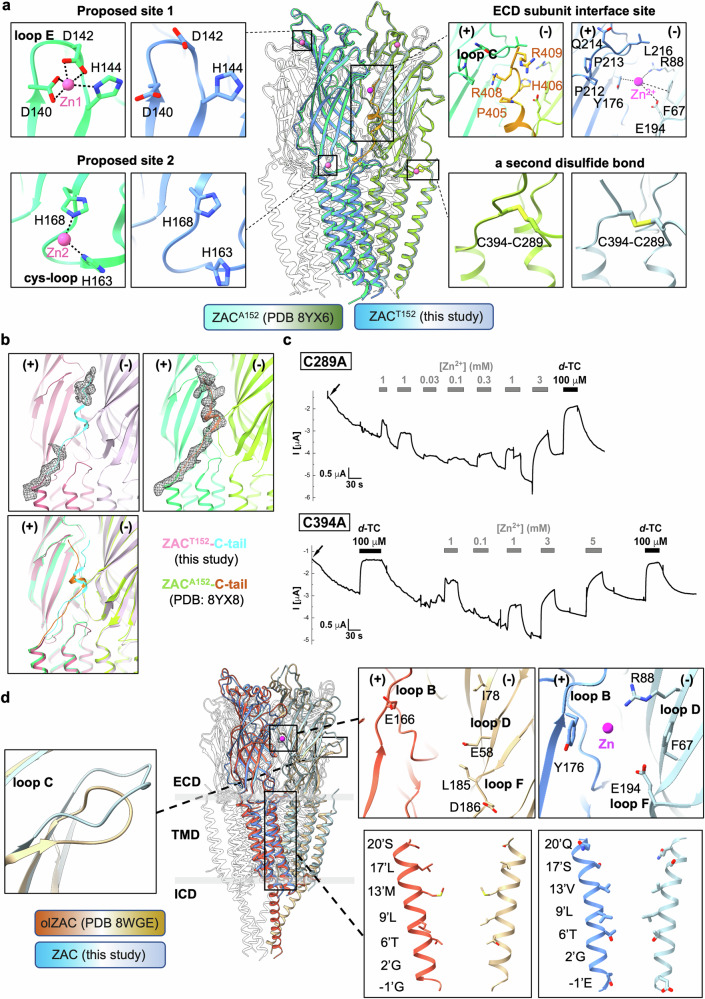
Comparison of ZAC structures. **a** Structural alignment of ZAC-Zn (blue, this study) and ZAC^A152^-Zn from a previous study^21^ (green, PDB: 8YX6) highlights detailed analyses of the Zn^2+^-binding site at the ECD subunit interface in ZAC-Zn and two proposed zinc-binding sites in ZAC^A152^-Zn^21^, with zinc ions shown as magenta spheres. The C-tail residues that interact with the ECD subunit interface in the ZAC^A152^-Zn structure^21^ are depicted as orange sticks. Both studies revealed a second disulfide bond between the C-tail and the M2–M3 loop. **b** Structural alignment of the ECD region in the ZAC-Apo model in this work (pale violet‒red) and the ZAC^A152^-Apo model^21^ (green, PDB: 8YX8); the C-tails are colored in cyan and orange, respectively (left). Density map of the C-tail in the ZAC-Apo structure from this work (middle) and in the ZAC^A152^-Apo structure^21^ (right). The principal subunit interface (+) and the complementary subunit interface (−) are colored differently. **c** Representative traces of the current responses evoked by various Zn^2+^ concentrations and *d-*TC (100 μM) in TEVC recordings of oocytes expressing C289A or C394A. The holding currents are given on the left axes, with arrows indicating when the oocyte was clamped. The holding currents in C289A- and C394A-expressing oocytes at the start of the recordings were consistently significantly lower than those in WT ZAC oocytes. Furthermore, in contrast to the stable holding currents in WT ZAC oocytes, the initial holding currents in these oocytes steadily decreased to lower levels during the recording time. Since C289A and C394A oocytes were incubated in MBS supplemented with *d-*TC (100 μM) from cRNA injection until the recordings, this decrease in holding current over time is likely a reflection of the dissociation of *d-*TC from C289A and C394A and further demonstrates the high levels of spontaneous activity of the two mutants. Both *d-*TC (100 μM) and Zn^2+^ (in a concentration-dependent manner) produced robust positive (upward) changes in the holding currents in the C289A and C394A oocytes. **d** A comparison between human ZAC-Zn (this study) and the *ol*ZAC^20^ (orange, PDB: 8WGE) reveals distinct features. These are illustrated in zoomed-in views of the orthosteric site, loop C region, and channel pore-lining residues of *ol*ZAC.

In all the structures, ZAC adopts the same pentameric structure as all the receptors in the CLR superfamily do. Each subunit features a large ECD consisting of ten β-strands with interconnecting loops and a transmembrane domain (TMD) composed of four α-helices (M1–M4) with interconnecting intracellular and extracellular loops, with the M2 helix lining the central channel. Two *N*-glycosylation sites (Asn99 and Asn170) are present in the ECD (Fig. 1b, c). In addition to the classical disulfide bond (Cys157-Cys171) that forms the prototypic Cys loop present in all CLRs, an additional disulfide bond is established between Cys289 in the extracellular M2–M3 loop and Cys394 at the C-terminus, and both of these two Cys residues are conserved throughout the mammalian ZAC homologs (Fig. 2a; Supplementary Fig. S2 and Table S2). To investigate the functional importance of this disulfide bond, alanine substitution mutants (C289A and C394A) were generated and characterized functionally in *Xenopus* oocytes via two-electrode voltage clamp (TEVC) electrophysiological recordings. Both mutants appeared to be efficiently expressed and trafficked to the cell membrane in the oocytes, and compared with WT ZAC, both exhibited very high levels of constitutive activity (Fig. 2c). These findings suggest that the Cys289-Cys394 disulfide bond is not essential for the proper folding or membrane trafficking of ZAC but is important for the conformational equilibrium of the channel.

The putative intracellular portals formed by helical amphipathic (HA) stretches in the M3–M4 loop are features of nAChRs and 5-HT_3_Rs, where they may function as important intracellular portals for ion conductance and rectification^23^. In contrast, human ZAC and other mammalian ZAC orthologs possess relatively short M3–M4 loops and lack such HA stretches, and the M3–M4 loop is absent in our cryo-EM maps (Fig. 1c; Supplementary Fig. S2).

Substantially larger density blobs were observed in the ZAC-Apo map at the ECD subunit interfaces than in the ZAC-Zn_partial_ and ZAC-Zn structures (Fig. 2b; Supplementary Fig. S3a). On the basis of a recent study showing that the C-tails occupy these sites in the human ZAC^A152^ variant structure^21^, we tentatively fitted a distal C-tail fragment into this discontinuous density. Compared with the ZAC^A152^ structure, the fitted fragment adopted a distinct orientation in the WT ZAC (ZAC^T152^)^21^ (Fig. 2b), suggesting that conformational dynamics are influenced by different conditions.

### Zinc recognition at the ECD subunit interface via cation‒π interactions

Closer inspection of the ZAC-Zn map and comparison with the ZAC-Apo map reveals that five additional densities corresponding to Zn^2+^ ions are positioned at all five interfaces between adjacent subunits in the ECD (Fig. 1a; Supplementary Fig. S3a). This location corresponds to that of the canonical orthosteric site targeted by the classical neurotransmitters in their cognate CLRs (i.e., nAChRs, 5-HT_3_Rs, GABA_A_Rs, and GlyRs). The Zn^2+^ ions in the ECD subunit interfaces of ZAC do not coordinate to typical zinc-coordinating residues such as cysteine and histidine in a tetrahedral network^18^, and their binding is primarily stabilized by two aromatic residues — Tyr176 in loop B in the principal subunit interface (+) and Phe67 in the β1 strand in the complementary subunit interface (−) — via cation–π interactions. Additional support for the coordination network seems to be provided by the side chains of Arg88 in loop D and of Glu194 in loop F to a lesser extent (Fig. 1d). Notably, these four residues that coordinate Zn^2+^ in a nearly planar arrangement are highly conserved in mammalian ZACs (Supplementary Fig. S2 and Table S2). Interestingly, while the poses and spatial positions of Phe67, Tyr176, and Glu194 in the ZAC-Apo and ZAC-Zn states are very similar, the side chain of Arg88 adopts a different pose in ZAC-Zn than in ZAC-Apo, with the latter pose closely resembling that of Arg88 in the ZAC^A152^-Apo structure^21^ (Supplementary Fig. S3b).

To confirm the coordination sphere for Zn^2+^ in ZAC, mutagenic analysis of these four key residues was conducted by determining the Zn^2+^ concentration relationships at WT and mutant receptors expressed in oocytes via TEVC recordings. In agreement with previous studies^13,19,22^, Zn^2+^ mediated robust concentration-dependent activation of WT ZAC in this expression system, with an EC_50_ of ∼0.3 mM (Fig. 1e, f; Supplementary Table S3). Alanine substitutions for Phe67 or Tyr176 led to ZAC mutants being completely non-responsive to Zn^2+^ concentrations up to 10 mM (Fig. 1e). Substitution of Phe67 or Tyr176 with histidine also resulted in non-responsive mutants (Supplementary Fig. S4 and Table S3), which was expected given that the His imidazole ring differs substantially from the aromatic ring of Phe/Tyr and that Zn^2+^ coordination to histidine typically requires a very specific structural arrangement in terms of metal ion–residue distances and angles^24^. Importantly, Zn^2+^ mediated concentration-dependent activation of the F67W and Y176W mutants, which highlights the importance of aromatic residues at these positions for zinc-evoked ZAC gating. Notably, Zn^2+^ displayed left-shifted and biphasic concentration–response relationships in these two mutants compared with that in WT ZAC, and the apparent channel properties of both mutants differed substantially from those of the WT receptor, as they exhibited faster desensitization and pronounced rebound currents at higher Zn^2+^ concentrations (Fig. 1f; Supplementary Fig. S4 and Table S3). Moreover, compared with WT ZAC, Y176W displayed substantially increased levels of spontaneous activity. Taken together, these findings suggest that the Phe/Tyr-for-Trp switch at these two positions not only lowers the energy barrier between the resting and active ZAC states but also impacts the desensitization characteristics of the channel. With respect to Arg88, both the removal of its long positively charged side chain (R88A) and substitution of it with a long aliphatic side chain (R88L) completely eliminated zinc activity at ZAC. Interestingly, Zn^2+^ exhibited a slightly left-shifted concentration–response relationship in a R88K mutant compared with that in WT ZAC, which not only suggests that a positively charged side chain at this position may be important for the zinc-mediated activation of ZAC but also that there may be some spatial flexibility in the position of Zn^2+^ at this site given the differences between the Arg and Lys side chains (Fig. 1e, f; Supplementary Fig. S4 and Table S3). In the case of Glu194, Zn^2+^ activated the E194A mutant in a seemingly concentration-dependent manner, but notably, the Zn^2+^-evoked responses through this mutant were characterized by very small (but significant) current amplitudes (Fig. 1e). The introduction of a large aliphatic substituent at this position (E194L) rendered ZAC completely unresponsive to Zn^2+^ (Supplementary Fig. S4 and Table S3). In notable contrast, Zn^2+^ evoked robust current responses through and exhibited a WT-like concentration‒response relationship in the E194Q mutant (Fig. 1f; Supplementary Fig. S4 and Table S3). This may indicate that the importance of the Glu194 carboxylate group for zinc-binding to and activating ZAC arises from the formation of a hydrogen bond with the metal ion rather than from an ionic interaction or, alternatively, that the elimination of a putative ionic interaction between Zn^2+^ and this Glu residue can be compensated for by the formation of hydrogen bonds with the amide group of the introduced Gln in E194Q. Finally, as important controls, several additional mutations of residues at both the principal interface (e.g., Gln214 and Leu216) and the complementary interface (e.g., Arg65, Asn148 and Leu178) — made as part of the mutagenic analysis of one of the *d*-TC binding sites in ZAC (see a later section) — were not found to substantially impact the ability of Zn^2+^ to activate ZAC or its agonist potency at the receptor (Supplementary Fig. S5 and Table S6). This finding suggests that the effects produced by mutations of Phe67, Arg88, Tyr167 and Glu194 are likely rooted in direct effects on Zn^2+^ binding.

In contrast to the ECD subunit interface binding site for Zn^2+^ in ZAC identified here, very different zinc-binding sites have recently been proposed on the basis of the *Oryzias latipes* ZAC (*ol*ZAC) and human ZAC^A152^ structures^20,21^. While the overall architecture of their Zn^2+^-bound ZAC^A152^ structure closely resembles that of ZAC^T152^-Zn in this work, with an root-mean-square deviation (RMSD) of 0.48 Å (Fig. 2a), Lu et al. proposed the presence of two zinc-binding sites located in loop E and in the Cys loop in the ZAC ECD, where Zn^2+^ is coordinated by the residues Asp140, Asp142 and His144 (site 1) and His163 and His168 (site 2), respectively^21^. The results from a previous elaborate mutagenic study probing almost all His, Glu and Asp residues in the ZAC ECD as putative Zn^2+^ coordinates (with Glu194 as one of the few exceptions) do not seem to support a substantial activation role for Zn^2+^ coordination to these sites^13^. With respect to the proposed site 1, Zn^2+^ exhibited WT-like or slightly right-shifted agonist concentration‒response relationships in ZAC mutants with mutations of Asp142 (D-to-A) and His144 (H-to-L/F)^13^. Although H120A or D140A/D142A/H144A ZAC mutants were non-responsive to Zn^2+^ (10 mM), proton-evoked ZAC signaling was also completely eliminated in these mutants (as was the case for several other mutants in the study), and this is more likely to reflect disruption of proper folding and/or expression of these ZAC mutants^13^. Alanine substitutions for His163 or His168 in the proposed site 2 yielded functional mutants that exhibited WT-like Zn^2+^ concentration‒response relationships^13^. The degree of evolutionary conservation of these two proposed sites across mammalian ZACs further indicates their involvement in Zn^2+^-mediated ZAC activation (Supplementary Fig. S2 and Table S2). While Asp140, Asp142 and His144 (D-D-H motif) in site 1 are fairly conserved across mammalian ZACs, several ZAC orthologs contain residues in these positions that are not considered Zn^2+^-coordination candidates, including the dog and naked-mole rat ZACs (G-D-H and F-D-Q motifs, respectively), whose Zn^2+^ concentration‒response relationships are comparable to those displayed by human ZAC^16^. The His168 residue is somewhat rare in mammalian ZACs, with Gln being the predominant residue in this position, including in the functional dog, horse, cow and naked-mole rat ZACs, and while His163 is highly conserved across mammalian ZACs, it is substituted for an Arg in the horse ZAC, which thus does not contain conventional zinc-coordinating residues in either of these proposed site 2 positions^16^ (Supplementary Fig. S2 and Table S2).

As for the four putative zinc-coordinating residues proposed by Jin et al.^20^ based on an *ol*ZAC apo structure, mutations of these four Asp and Glu residues in *ol*ZAC resulted in only modest effects on Zn^2+^ potency^20^. The four residues display different degrees of conservation across mammalian ZACs (Fig. 2d; Supplementary Fig. S2 and Table S2). Specifically, in human ZAC, only one of these four residues is pseudo-conserved, and alanine substitution for this residue (Glu113) results in a functional mutant with WT ZAC-like Zn^2+^ properties^13^. Notably, *ol*ZAC has very low amino acid sequence homology (< 24% identity) with human ZAC and other mammalian ZACs and thus is just as homologous to 5-HT_3_R subunits as it is to ZAC. Thus, even though *ol*ZAC is activated by Zn^2+^, it seems relevant to consider whether it is an ortholog of human ZAC and to what extent it represents a source for information in regard to the mammalian ZACs.

While the identified zinc-binding site in the five ECD subunit interfaces of ZAC aligns with the location of the orthosteric sites in the classical CLRs, closer inspection of the structural architecture of the site in ZAC reveals some notable differences (Supplementary Fig. S7). A typical binding component involving the loop C region commonly observed in neurotransmitter–CLR interactions is not present in ZAC, and moreover, no obvious differences in the positions of this loop are observed between the ZAC-Apo and ZAC-Zn structures (Supplementary Fig. S8). This may be due to the relatively short length of the loop C region in ZAC and/or the smaller size of Zn^2+^ as an agonist than those of ACh, 5-HT, GABA, and glycine, which bypass the involvement of loop C in direct interactions with the metal ion (Supplementary Figs. S7, S8). The fact that substitutions of loop C residues Gln214 and Leu216 with relatively small residues (Q-to-A/K, L-to-A) have negligible effects on Zn^2+^ agonist potency, whereas the introduction of larger residues at these two positions (Q-to-L/W, L-to-R) renders ZAC non-responsive to Zn^2+^, could indicate that loop C does not contribute directly to zinc binding to ZAC but is in proximity to it (Supplementary Fig. S5 and Table S6). These structural features underscore the unique agonist coordination environment in ZAC compared with those in other CLR superfamily members.

### Ion permeation path

**Fig. 3:**
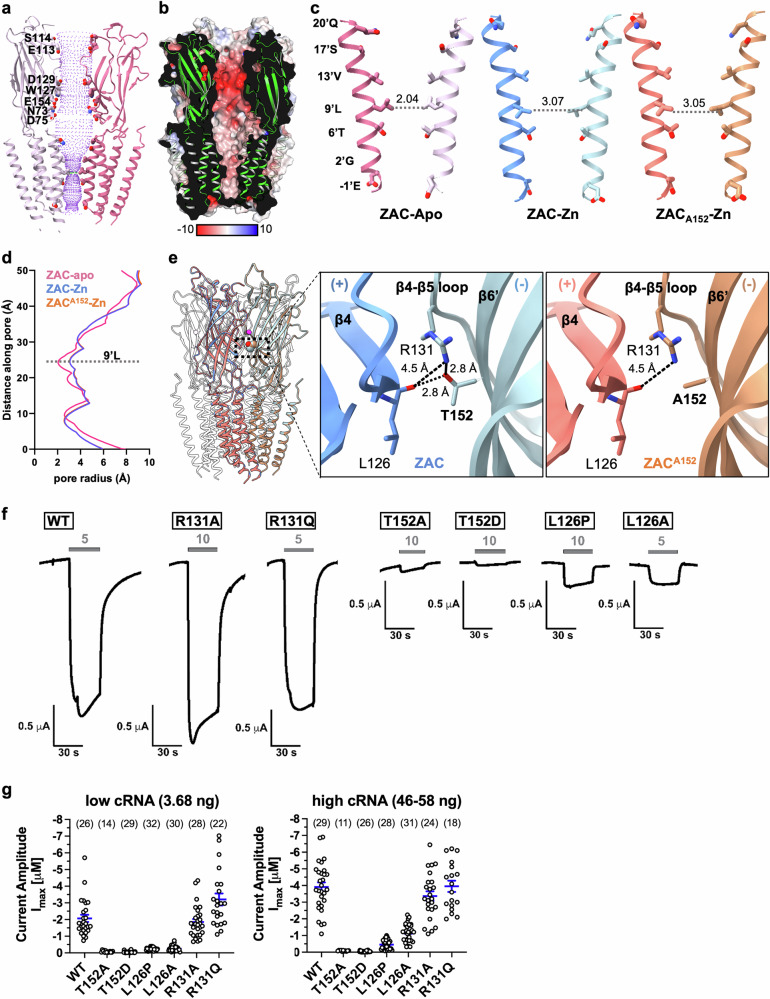
Ion permeation pathway of ZAC and ZAC^A152^ variant structures. **a** Global view of the ion permeation pathway. Two ZAC subunits are illustrated as ribbons, with key ECD residues lining the permeation pathway depicted as sticks. The solvent-accessible surface is represented by purple dots. **b** The solvent-accessible electrostatic potential is mapped onto the ZAC surface, color-coded from −10 kT^e-1^ to +10 kT^e-1^, ranging from red (negative) to blue (positive). **c** Depictions of the channel pore in the ZAC-Apo (left), ZAC-Zn (middle) and ZAC^A152^-Zn (right) structures. Exposed side chains are depicted as sticks, with dashed lines indicating the radius of the 9’L constriction (in Å). The principal subunit interface (+) and the complementary subunit interface (−) are color-coded in dark (+) and light (−) intensities for clarity. **d** Pore profiles for the ZAC-Apo (magenta), ZAC-Zn (blue) and ZAC^A152^-Zn (orange) structures in the TMD region are shown, illustrating the differences in pore size and shape across these states. **e** Left, structural alignment of ZAC-Zn (T152) (blue) and ZAC^A152^-Zn (orange). The residues at position 152 are highlighted as spheres and emphasized with a black frame. The zinc ion is shown as a magenta ball. Right, detailed views of the network interactions at the T152 (ZAC-Zn) and A152 (ZAC^A152^) residues are shown, with interactions represented by dashed lines. **f** Examples of traces of the current responses evoked by Zn^2+^ (5 mM or 10 mM) in oocytes expressing WT ZAC and ZAC mutants R131A, R131Q, T152A, T152D, L126P and L126A in TEVC recordings. All traces shown are from recordings performed two days after the oocytes were injected with 3.68 ng of WT or mutant ZAC cRNA. **g** I_max_ evoked by Zn^2+^ at WT ZAC and mutant T152A, T152D, L126P, L126A, R131A, and R131Q ZACs in oocytes in TEVC recordings two days after injections of “low” (3.68 ng) and “high” (46–58 ng) cRNA quantities. The specific “high” cRNA quantities injected into the oocytes were as follows: WT, 46 ng; T152A, 51 ng; T152D, 54 ng; L126P, 48 ng; L126A, 58 ng; R131A, 54 ng; and R131Q, 51 ng. The I_max_ are the I_max_ values based on the fitted concentration‒response curves or the highest current amplitudes evoked by 3, 5, and 10 mM Zn^2+^ in the oocytes. The *n*-number (the number of recorded oocytes) for each receptor is given in parentheses above the data.

The ZAC structures reveal a pentameric complex with a wide extracellular vestibule, approximately 20 Å in width and 50 Å in height. The surface of this vestibule is highly electronegative and is formed by residues Asn73, Asp75, Glu113, Ser114, Asp129, and Glu154 from both adjacent subunits forming the interface, which likely contributes to the attraction of hydrated cations (Fig. 3a, b). Following the ECD, the TMD tapers toward the intracellular side, forming the channel pore, which is lined by residues Glu (−1’), Gly263 (2’), Thr267 (6’), Leu270 (9’L), Val274 (13’), Ser278 (17’), and Gln281 (20’) on each of the M2 helices (Fig. 3c).

Consistent with its ability to pass Na^+^ and K^+^ ions, the channel is electronegatively charged. Between residues 9’L and 16’S, the hydrophobic region of the TMD forms a constriction, acting as a hydrophobic gate, an essential and highly conserved feature in CLR family members^25^. In the ZAC-Apo state, this gate, formed by the 9’L residues, represents the narrowest part of the channel, with a radius of 2.04 Å. This constriction at 9’L is also observed in the resting states of 5-HT_3A_R^26^ and α7 nAChR^27^, with radii of 2.3 Å and 1.2 Å, respectively. In the ZAC-Zn structure, the gate opens slightly to a radius of 3.07 Å (Fig. 3d). Analogous to the effects of mutations of 9’L in classical CLRs^28–31^, ZAC^L9’X^ mutants exhibit substantially different channel properties than WT ZAC does, including a slower deactivation process, which reflects that the ‘leucine ring’ formed by the five 9’L residues plays a crucial role in stabilizing the resting ZAC conformation^13^. Additionally, both ends of the channel pore are lined with polar/negative residues: 20’Q on the extracellular side and −1’E on the intracellular side. This configuration is similar to the 20’E, −1’E and 20’D, −1’E arrangements in the α7 nAChR and the 5-HT_3_AR, respectively (Supplementary Fig. S8c), thus constituting a crucial region for ion selectivity.

### Structural basis for the functional consequences of high-frequency variation

The single-nucleotide polymorphism (SNP) c.454 G>A in the *ZACN* gene encodes a non-synonymous variant (Thr152Ala, ZAC^A152^), which is found at such high allele frequencies in all ethnicities that any distinction between major and minor alleles in the general population becomes somewhat academic^22^. In TEVC recordings from *Xenopus* oocytes, compared with WT ZAC (ZAC^T152^), homopentameric ZAC^A152^ exhibits dramatically reduced Zn^2+^-evoked current amplitudes^22^, suggesting a key impact of the identity of this residue on ZAC gating. To explore the structural basis for these functional implications, a 2.63 Å resolution map of the ZAC^A152^ variant in the presence of Zn^2+^ was resolved (ZAC^A152^-Zn). The overall architecture of the structure closely resembles that of ZAC-Zn, with clear densities for Zn^2+^ ions observed at the five ECD subunit interfaces (Supplementary Fig. S3a).

The Thr/Ala152 residue is located at the bulge point of the long β6’ strand preceding the prototypic Cys loop in the ECD, from which it projects into the subunit interface toward the neighboring subunit. In ZAC-Zn, the hydroxy group in the side chain of Thr152, supported by the guanidinium group of Arg131 on the same complementary (−)-face of the interface, interacts with the main chain carbonyl oxygen of Leu126 on the β4 strand of the principal (+)-face (Fig. 3d; Supplementary Fig. S3c). This intricate network forms a potential fulcrum point at the narrowest distance between two adjacent subunits and is strategically positioned between the agonist (Zn^2+^) binding site and the TMD. In ZAC^A152^-Zn, this interaction network is weakened (Fig. 3d), potentially dampening the transmission of momentum from agonist binding to channel pore opening. In support of this interaction network, the introduction of Ser or Val at position 152 produces receptors with intermediate maximal current amplitudes (I_max_) compared with those of WT ZAC and ZAC^A152^, whereas an Ile or a Phe residue at this position is just as unfavorable as Ala^22^. Although admittedly speculative, the presence of several hydrophobic residues in the surroundings of residue 152 may explain why a Thr, given the additional methyl group in its side chain, would be superior to a Ser in this position and why Val, as a bulkier aliphatic residue, would be superior to Ala.

To further investigate the functional role of the interaction network centered on Thr152, we characterized the Zn^2+^ concentration‒response relationships and I_max_ in six mutants (R131A, R131Q, L126P, L126A, T152D and T152A) expressed in oocytes by TEVC recordings (Fig. 3f, g; Supplementary Table S4). The agonist potencies and I_max_ exhibited by Zn^2+^ in the R131A and R131Q mutants did not differ significantly from those in WT ZAC, which suggests that Arg131 is not critically involved in the network. Zn^2+^ exhibited WT-like concentration‒response relationships at L126P and L126A, but the Zn^2+^-evoked I_max_ in oocytes expressing these mutants were markedly reduced compared with those evoked through WT ZAC (Fig. 3f, g; Supplementary Table S4). The effect of the Leu-for-Pro substitution is consistent with the disruption of the local backbone conformation by the introduced Pro, thus impacting the main chain carbonyl oxygen forming the interaction with Thr152. As the L126A mutation would not be expected to mediate structural constraints on backbone geometry, the sizeable reduction in I_max_ produced by this substitution suggests that the bulky side chain of Leu126 may play an important role in the precise orientation of the backbone or in the local environment of this network. The original goal behind the T152D mutation was to determine whether a negatively charged side chain of the residue at this position could potentially form a stronger interaction with the positively charged side chain of Arg131 than that formed between T152 and R131 in WT ZAC. However, the T152D mutation was found to be just as detrimental for Zn^2+^-evoked current amplitudes through ZAC as T152A (Fig. 3f, g; Supplementary Table S4). It is possible that the D152 carboxylate group is not positioned close enough to the guanidium group of R131 to establish an ionic interaction in T152D. However, considering that Arg131 does not seem to be essential for this network, the dramatic effect of the T152D mutation on ZAC functionality is more likely rooted in Asp not being able to establish the same interaction with the main chain carbonyl oxygen of Leu126 as Thr152 in WT ZAC. Collectively, these functional data thus identify Leu126 and Thr152 as key contributors to this intermolecular network in ZAC, which seems to be essential for its functionality. Notably, analogous to this network, interactions between residues in the β6’ strand and in the β4–β5 strands on adjacent complementary and principal faces of subunit interfaces, respectively, have been proposed to be involved in this coupling of agonist binding to channel gating in the α7 nAChR^32^.

### The selective antagonist TTFB targets a site in the channel pore

TTFB has previously been identified as a relatively potent and selective ZAC inhibitor, with an IC_50_ of approximately 3 µM^19^. Mechanistic studies of TTFB at WT ZAC and 5-HT_3A_R/ZAC and ZAC/α1-GlyR chimeras have indicated that the inhibitor acts allosterically, most likely through a site in the TMD or ICD of the receptor^19^. To elucidate the structural basis of TTFB-mediated ZAC inhibition, we determined a TTFB-bound ZAC structure at 2.54 Å resolution. Comparison with the ZAC-Apo structure revealed additional densities within the channel, which were modeled as TTFB molecules, thus providing detailed insights into the binding mode of the ligand in these regions (Supplementary Fig. S3e).

**Fig. 4:**
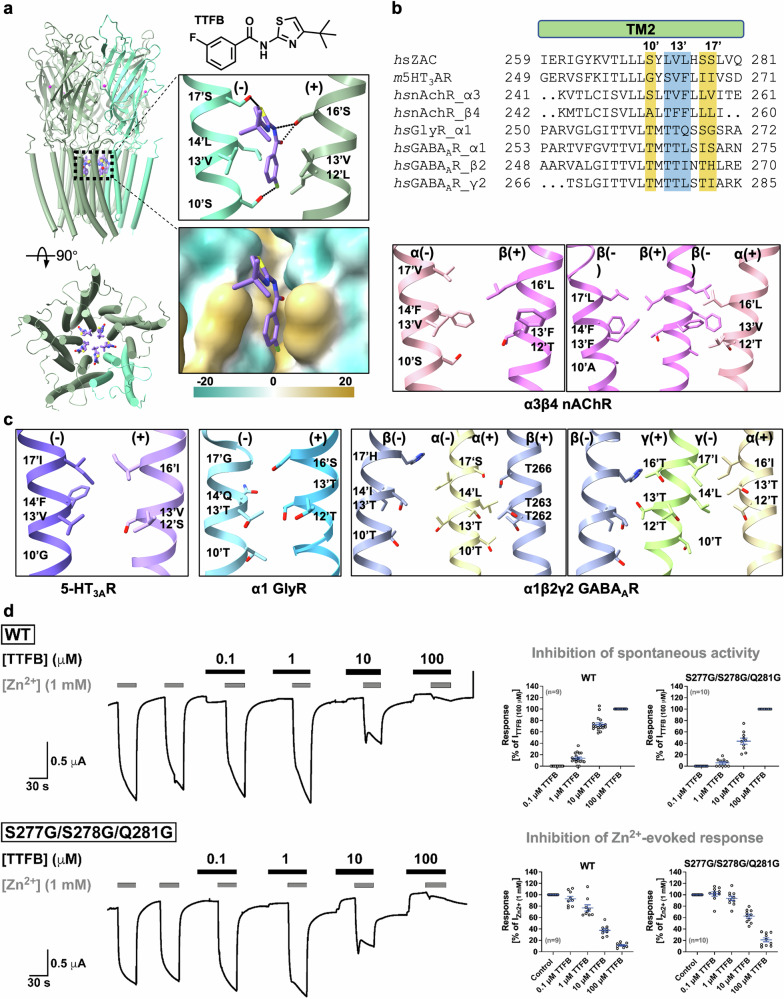
Selective binding of the antagonist TTFB to the ZAC TMD. **a** The structure of ZAC bound to the selective antagonist TTFB is viewed from the membrane plane (top) and top-down (bottom), with transmembrane helices represented as tubes and TTFB depicted as purple sticks. A detailed view of the TTFB-binding site is shown on the right, where the hydrogen bonds are indicated by dashed lines. The principal subunit interface (+) and the complementary subunit interface (−) are colored for distinction. Below that, the binding pocket is represented as a solid surface, color-coded by hydrophobicity (range, −20 to 20, from hydrophilic to hydrophobic), highlighting the hydrophobic and polar regions involved in the binding interaction. **b** Sequence alignment of M2 helices across selected CLRs. Residues in ZAC involved in the hydrophobic interactions with TTFB are highlighted with a blue shade, whereas residues forming polar interactions are given with an orange shade. *hs*, *Homo sapiens* (human); *m*, mouse. **c** The structural landscape of the TTFB-binding site in ZAC (Fig. 3a) compared with similar regions across other selected CLRs, i.e., 5-HT_3A_R (PDB: 6DG8), α3β4 nAChR (PDB: 6PV7), α1 GlyR (PDB: 4X5T), and α1β2γ2 GABA_A_R (PDB: 6D6T), emphasizing the distinct binding pocket for TTFB in ZAC. **d** Antagonist properties displayed by TTFB in WT ZAC and S277G/S278G/Q281G ZAC. Left, representative traces of the current responses in TEVC recordings evoked by Zn^2+^ (1 mM) in the absence and presence of TTFB (0.1, 1, 10, and 100 μM) through WT and S277G/S278G/Q281G ZAC expressed in oocytes. Right, TTFB-mediated antagonism of the spontaneous activity of (top) and the Zn^2+^ (1 mM)-evoked response through (bottom) WT and S277G/S278G/Q281G ZAC expressed in oocytes in TEVC recordings. Data for the inhibition of spontaneous activity represent positive (upward) changes in holding currents produced by TTFB during the 30 s preincubation and are normalized to the positive (upward) change in holding currents produced by TTFB (100 μM) [I_TTFB(100 μM)_] in the oocytes. Data for the inhibition of the Zn^2+^ (1 mM)-evoked response represent the reduced inward currents produced by the subsequent co-application of Zn^2+^ (1 mM) and TTFB and are normalized to the current produced by Zn^2+^ (1 mM) alone [I_Zn_^2+^_(1 mM)_] in the oocytes. Data are given as the individual data points with mean ± SEM values. Data for all TTFB site mutants are given in Supplementary Fig. S9 and Table S5.

In the ZAC-TTFB structure, five TTFB molecules are located between adjacent M2 helices near the extracellular side of the channel (Fig. 4a). All five ECD subunit interfaces in the structure are occupied by Zn^2+^ ions, in agreement with the proposed allosteric mechanism of the inhibitor^19^. We noted that at the same Zn^2+^ concentration used to obtain the ZAC-Zn/TTFB structure, only one Zn^2+^ site is readily occupied in the ZAC-Zn_partial_ structure, which may represent a more resting-like conformation than ZAC-Zn/TTFB. The exact structural basis for this difference is not fully clear. One putative explanation is that the wedging of TTFB between the adjacent M2 helices in the TMD may induce a conformational change that is propagated back to the ECD subunit interfaces, thereby facilitating Zn^2+^ binding to these at lower concentrations. In its binding, TTFB interacts with a hydrophobic pocket formed by residues 12’L and 13’V on (+)-M2 and 13’V and 14’L on (−)-M2. Additional receptor‒ligand interactions include the formation of hydrogen bonds between the hydroxy groups of (−)-10’S, (−)-17’S, and (+)-16’S and the fluoro, sulfur, and carboxyl oxygen atoms of TTFB, respectively (Fig. 4a). In the other cation-selective CLRs, the 5-HT_3_Rs and nAChRs, this hydrophobic pocket is sterically hindered because of the presence of Phe residues at the 12’, 13’ and 14’ positions, and several of the polar residues coordinating to TTFB in ZAC (10’, 16’, 17’) are also not conserved in these receptors (Fig. 4b, c). Conversely, the anion-selective CLRs, the GlyRs and GABA_A_Rs, possess more polar pockets (12’, 13’, and 14’), which would be suboptimal for TTFB interaction. These structural distinctions may underscore the selective antagonism mediated by TTFB at ZAC versus other CLRs.

To assess the contributions of these residues to TTFB binding, we performed TEVC recordings in oocytes expressing various ZAC mutants. We evaluated the effects of the mutations on both the TTFB-induced block of spontaneous ZAC activity (reflected in the positive (upward) change in holding current produced by TTFB during a 30 s preincubation) and of Zn^2+^-evoked ZAC currents (reflected in the reduced inward current elicited during the subsequent co-application of Zn^2+^ (1 mM) and TTFB). While TTFB displayed WT-like antagonist properties in the S277A (16’S→A) mutant, it exhibited slightly (∼2-fold) reduced antagonist potencies in its inhibition of S278A (17’S→A) and S277G/S278G/Q281G (16’S/17’S/20’Q→G/G/G) mutants compared with WT ZAC (Fig. 4d; Supplementary Fig. S9 and Table S5). Despite considerable efforts, reliable antagonist properties for TTFB could not be determined for mutants comprising alanine substitutions for the three residues forming its hydrophobic pocket in ZAC. Receptors assembled from L273A (12’L→A) or V274A (13’V→A) in the oocytes were highly constitutively active (judging from the very negative holding currents of these oocytes). Even when the L273A and V274A oocytes were incubated in the presence of 100 μM *d-*TC from cRNA injection until the recordings (in an attempt to alleviate the impact of this constitutive activity on the oocytes), they were consistently of very poor quality on the day of recording (soft, leaky, and often ruptured immediately following clamping), which rendered recordings infeasible. Although L275A (14’L→A) ZAC appeared to be less constitutively active, oocytes expressing this mutant were also of very poor quality on the day of recording, and the functional properties determined for Zn^2+^ and TTFB in this mutant varied dramatically from oocyte to oocyte and were thus not reproducible (data not shown). Thus, in light of the notable but relatively modest reductions in TTFB antagonist potency induced by the S278A and S277G/S278G/Q281G mutations and our inability to elucidate the putative importance of the L273, V274, and L275 residues for ZAC inhibition, we cannot provide clear and interpretable functional data supporting the molecular basis for TTFB binding to ZAC.

Although allosteric modulation of CLRs through binding sites residing in their TMDs is a shared mechanism for a range of different modulators, the location of these sites in this domain and the interaction patterns of modulators and receptors here vary significantly. For instance, the allosteric agonist ivermectin binds to GluCl between the M2 and M3 helices of the (+)-subunit and the M1 helix of the (−)-subunit via hydrogen bonds and extensive hydrophobic contacts (PDB: 3RIF)^33^. In addition to their high-affinity ECD subunit interface-binding site, diazepam and other benzodiazepines modulate GABA_A_Rs by interacting with the M2 and M3 helices of the (+)-subunit and the M1 helix of the (−)-subunit (PDB: 6HUP)^34^, a binding cavity also targeted by numerous other modulators^35–39^. Estradiol binds the ρ1 GABA_A_R at the ECD–TMD interface between adjacent subunits, interacting with loop F and the M2–M3 loop on the (+)-subunit, as well as the β1–β2 loop and M2–M3 loop on the (−)-subunit via hydrophobic and π–orbital interactions (PDB: 8RH7)^40^ (Supplementary Fig. S10). To our knowledge, the allosteric site targeted by TTFB between adjacent M2 helices in ZAC is novel and thus further illustrates the sensitivity of CLR signaling to small-molecule ligands targeting various sites in the TMD.

### Orthosteric and allosteric inhibition by *d*-TC

**Fig. 5:**
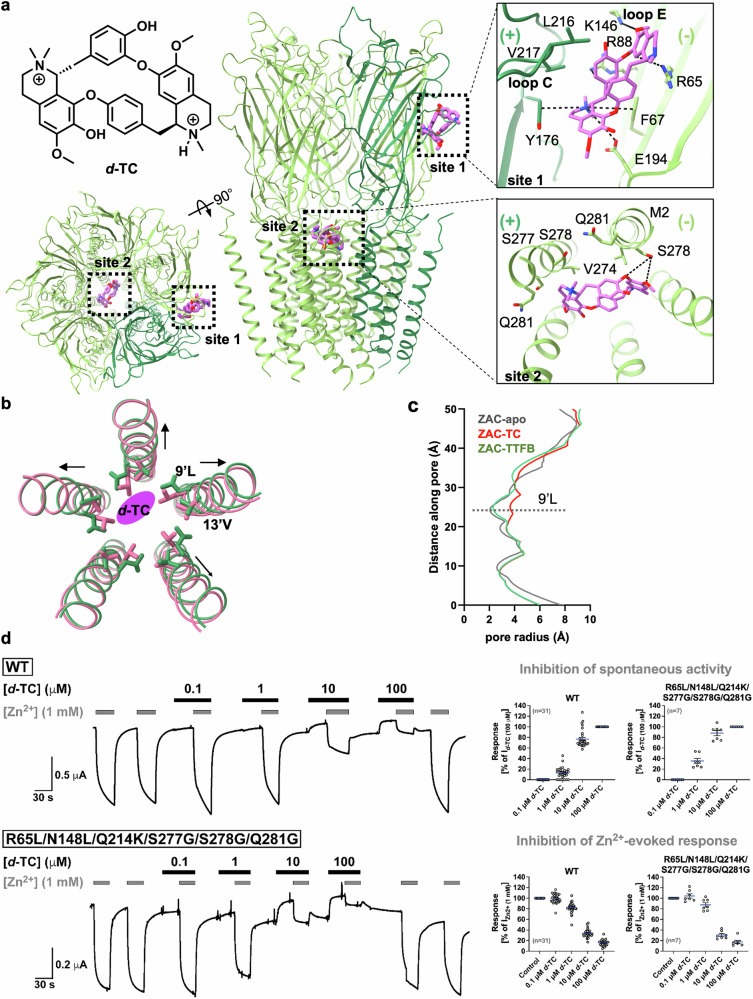
Orthosteric and allosteric *d*-TC-binding sites in ZAC. **a** Structure of ZAC bound to the non-selective inhibitor *d*-TC. Side view (middle) and top view (bottom left) of the ZAC-*d*-TC complex showing the two distinct binding sites, site 1 and site 2, with the chemical structure of *d*-TC shown on the top left. A detailed view of the interactions of *d*-TC with ZAC at the two sites is depicted on the right, with dashed lines representing key hydrogen bonds and cation‒π interactions. **b** Superposition of the M2 helices from ZAC-Apo (colored pale violet red) and ZAC-*d*-TC (colored green) illustrates the conformational changes induced by *d*-TC binding. The side chains of residues 9’L and 13’V are shown as sticks, highlighting the subtle yet significant changes in the TMD upon *d*-TC binding. The *d*-TC molecule is shown as an orchid oval, and the RMSD between the two structures is 0.074, suggesting minimal yet crucial movement in the TMD. **c** Pore profile comparison is shown for ZAC-Apo (in gray), ZAC-*d*-TC (in red) and ZAC-TTFB (in green) for the TMD region. **d** Antagonist properties displayed by *d*-TC in the WT and in a sextuple ZAC mutant with triple mutations at both site 1 and site 2. Left, representative traces of the current responses evoked by Zn^2+^ (1 mM) in the absence and presence of *d*-TC (0.1, 1, 10, and 100 µM) through WT and R65L/N148L/Q214K/S277G/S278G/Q281G ZAC expressed in oocytes in TEVC recordings. Right, *d*-TC-mediated antagonism of the spontaneous activity and of the Zn^2+^ (1 mM)-evoked response of WT ZAC and R65L/N148L/Q214K/S277G/S278G/Q281G expressed in oocytes in TEVC recordings. Data for the inhibition of the spontaneous activity represent positive (upward) changes in the holding current produced by *d*-TC during the 30 s preincubation and are normalized to the positive (upward) change in the holding current produced by *d*-TC (100 μM) (I_*d*-TC__(100__μM)_) in the oocytes. Data for the inhibition of the Zn^2+^ (1 mM)-evoked response represent the reduced inward currents produced by the subsequent co-application of Zn^2+^ (1 mM) and *d*-TC and are normalized to the current produced by Zn^2+^ (1 mM) alone (I_Zn_^2+^_(1 mM)_) in the oocytes. Data are given as the individual data points with mean ± SEM. The data for all the *d*-TC site 1 and site 2 mutants are given in Supplementary Figs. S5 and S6 and Table S6.

The curare alkaloid *d*-TC is a relatively promiscuous inhibitor of cation-selective CLRs, targeting both nAChRs and 5-HT_3_Rs^41–43^, and prior to the development of safer modern neuromuscular blocking agents, has been applied as a nondepolarizing skeletal muscle relaxant during surgery^44^. *d*-TC also acts as a relatively potent ZAC inhibitor, with an IC_50_ of ∼3 µM for the receptor^15,22^. The *d*-TC-bound ZAC structure, resolved at 2.97 Å resolution, exhibits an overall architecture similar to that of the ZAC-Apo structure. Despite the relatively low quality of local densities, two *d*-TC molecules were identified as occupying distinct binding sites: a binding site at the ECD subunit interface (site 1) and a site within the channel pore (site 2) (Fig. 5a; Supplementary Fig. S2d).

At site 1, the quaternary ammonium group of *d*-TC mimics the positive charge of the Zn^2+^ ion, forming cation‒π interactions with Tyr176 in the (+)-subunit and Phe67 in the (−)-subunit, with additional cation‒π interactions occurring between Arg65 and Arg88 in the (−)-subunit and the aromatic rings of *d*-TC. Lys146 on (−)-loop E forms a hydrogen bond with the oxygen atom of *d*-TC. *d*-TC binding is also stabilized by the residues Gln214, Leu216 and Val217 on (+)-loop C (Fig. 5a) and by Leu123, Asn148, Leu178, and Glu194 to a lesser extent. While the binding of a *d*-TC molecule is only defined to one of the ECD subunit interfaces, theoretically, the inhibitor could occupy any or all of these sites in the pentameric ZAC. This mirrors the variable binding site occupancy observed for *d*-TC in the *Aplysia californica* AchBP structure (PDB: 2XYT)^41^, where *d*-TC adopts at least three different binding orientations that are all characterized by primarily forming cation‒π interactions with conserved aromatic residues in the binding pocket^41^. *d*-TC also targets the ECD subunit interface site in heteropentameric nAChRs, for example, in the *Torpedo californica* (α)_2_βγδ nAChR (PDB: 7SMS), where *d*-TC binds at the α-γ and α-δ interfaces, engaging primarily in cation‒π interactions with the aromatic box in both^45^ (Supplementary Fig. S11).

At site 2, *d*-TC binds to the extracellular entry of the ZAC channel, exhibiting a pore-blocking pattern similar to that observed in the *d*-TC-bound (α)_2_βγδ nAChR structure^45^, albeit with slight differences in orientation. In ZAC, *d*-TC is wedged by residues 13’V (Val274), 16’S (Ser277), and 20’Q (Gln281) and forms a hydrogen bond with the hydroxy group of 17’S (Ser278) on one of the M2 helices, oriented nearly parallel to the channel axis. In contrast, the *d*-TC in the nAChR structure is positioned more perpendicular to the channel axis, but the overall interaction network is similar to that in ZAC-*d*-TC. Notably, the fourth binding site of *d*-TC observed in the *d*-TC-nAChR complex (at the extracellular ends of the M1, M3, and M4 helices) is absent in the ZAC structure (Supplementary Fig. S11).

Collectively, the two *d*-TC molecules stabilize ZAC in a desensitized-like conformation that resembles that of the *d*-TC-bound nAChR, where the 9’L ring (radius, 3.64 Å) is more open than in the apo state (2.04 Å) and the TTFB-bound state (2.40 Å) (Fig. 5b, c). In the *d*-TC-bound ZAC structure (ZAC-*d*-TC), the M2 helix expands, causing the channel pore to widen at 9’L. This observation is consistent with the mechanism of many non-competitive antagonists, where binding at distinct sites within the same receptor subunits stabilizes the channel in a resting/desensitized state, effectively blocking the channel through a steric mechanism^46^. Thus, these findings further support the non-selectivity of *d*-TC in its targeting of channel pore sites in various CLRs while also highlighting the diversity in its binding patterns observed across different homologous receptors.

The two *d*-TC-binding sites in the ZAC-*d*-TC structure were probed by mutagenic analysis, where the effects of the mutations on the *d*-TC-mediated block of both spontaneous ZAC activity (reflected in the positive (upward) change in the holding current produced by *d*-TC during a 30 s preincubation) and Zn^2+^-evoked ZAC currents (reflected in the reduced inward current produced in the subsequent co-application of Zn^2+^ (1 mM) and *d*-TC) in the oocytes were assessed (Fig. 5d). In agreement with previous findings in both mammalian cells and oocytes^15,22^, *d*-TC inhibited both the spontaneous activity of and the Zn^2+^ (1 mM)-evoked currents through WT ZAC in a concentration-dependent manner, with IC_50_ values of ∼3 μM (Fig. 5d; Supplementary Figs. S5, S6 and Table S6).

With respect to site 1, the non-responsiveness of the F67A, R88A, and Y176A mutants to Zn^2+^-mediated activation (Fig. 1e) ruled out investigations of the contributions of these residues to *d*-TC binding. Thus, our investigation of this site focused on 7 other residues either directly involved in *d*-TC binding or lining its binding pocket. Substitutions of Leu123 or Val217 for alanine yielded mutants that consistently produced poor-quality oocytes not suited for TEVC recordings. Although interesting, we will here refrain from speculating about whether this could arise from effects of these mutations on ZAC function. In contrast to these mutants, several mutations of Arg65 (R-to-A/L/W), Asn148 (N-to-A/L/W) and Leu178 (L-to-A/K) on the (−)-side and of Gln214 (Q-to-A/K) and Leu216 (L-to-A) on the (+)-side of the ECD subunit interface yielded functional mutants that exhibited Zn^2+^ EC_50_ values either similar to or slightly higher (up to 2.5-fold) than those displayed by WT ZAC (Supplementary Table S6). This enabled us to use Zn^2+^ (1 mM), constituting an EC_60_–EC_90_ concentration at the WT and mutant ZACs, as the agonist concentration for these experiments. Surprisingly, *d*-TC exhibited very similar antagonist potencies in all these mutants as those in the WT ZAC, both in terms of both its inhibition of spontaneous activity and the Zn^2+^-induced response. Moreover, combinations of some of these mutations in R65L/N148L and R65L/N148L/Q214K mutants did not reduce the antagonist potency of *d*-TC compared with that in WT ZAC (Supplementary Fig. S5 and Table S6).

Our mutagenic analysis of site 2 focused on three of the four residues coordinating to *d*-TC at this site, as it was impossible to record from oocytes expressing the V274A mutation (outlined above). Mutations of Ser277 (S-to-A), Ser278 (S-to-A), and Gln281 (Q-to-G/L/K) produced functional mutants that all exhibited essentially WT-like Zn^2+^ concentration‒response relationships (although several of these mutants exhibited increased spontaneous activity compared with that of the WT), whereas the introduction of larger substituents at positions 277 and 278 resulted in non-expressed or non-functional receptors (Supplementary Table S6). *d*-TC was found to be an equipotent antagonist of all these mutants as of WT ZAC. The greatest reduction in antagonist potency observed for any of these mutants was the 2.5-fold increase in the IC_50_ value exhibited by *d*-TC for Q281K (7.6 μM vs 3.0 μM for WT ZAC), which we do not consider to be biologically important. Strikingly, elimination of the side chains of all three residues in the S277G/S278G/Q281G mutant did not significantly attenuate the antagonist potency displayed by *d*-TC (Supplementary Fig. S6 and Table S6).

We considered whether the apparent dual-action mechanism underlying *d*-TC-mediated ZAC inhibition could explain the lack of substantial effects of any of the site 1 and site 2 mutations on its activity. We hypothesized that if *d*-TC binds to these two sites with comparable affinities and if the *d*-TC occupation of one of them is sufficient for ZAC inhibition, then elimination or impairment of *d*-TC binding to either of the sites would not necessarily impact its antagonist potency. To test this hypothesis, we combined site 1 mutations R65L/N148L/Q214K and site 2 mutations S277G/S278G/Q281G in a mutant that analogously to the site 1 and site 2 triple mutants, exhibited WT-like Zn^2+^ properties. However, also analogously to its two “parental” mutants, the antagonist potency displayed by *d*-TC for the sextuple mutant R65L/N148L/Q214K/S277G/S278G/Q281G was similar to that for WT ZAC (Fig. 5d; Supplementary Figs. S5, S6, and Table S6).

## Discussion

The transition metal zinc has garnered increasing attention as a transmitter, in addition to its well-established structural and catalytic roles in proteins that facilitate multiple physiological processes. ZAC is an understudied member of the CLR superfamily, and its importance as an in vivo zinc sensor for zinc biology in mammals is unknown. In this work, we elucidate how ZAC is activated by Zn^2+^, identify a structural network likely responsible for the marked differences in functionality between WT ZAC and its high-frequency variant, and provide detailed insights into the distinct mechanisms underlying inhibition mediated by two antagonists.

On the one hand, the identification of the zinc-binding site in ZAC in this study confirms that ZAC, analogously to its CLR family members, is activated via the canonical ECD subunit interface site in these receptors. On the other hand, even though the essential contributions of cation‒π interactions to this binding also align with the key roles of aromatic residues in agonist binding to classical CLRs, this binding mode is somewhat anomalous for this particular agonist. Owing to its redox-inert nature, zinc is typically coordinated by the sulfur atom of cysteine, the imidazole nitrogen atoms of histidine, and the carboxylate groups of aspartate and glutamate in tetrahedral arrangements, with distinct coordination numbers and binding geometries^24^. However, although relatively rare in proteins, cation‒π interactions between Zn^2+^ and its coordinating residues have been observed in Zn^2+^–arene and Zn^2+^–halobenzene organic complexes^47–49^. We propose that the coordination environment observed for Zn^2+^ in ZAC, which is primarily formed by the aromatic residues Phe67 and Tyr176 with additional support from the more distant Glu194 and Arg88 residues in a nearly planar arrangement, is strongly supported by the mutational analysis of these four residues. Moreover, the pronounced evolutionary conservation of these four residues, including the aromatic residues, across mammalian ZACs further supports this. The alignment of human ZAC with 88 non-human mammalian ZACs revealed that Phe67 is conserved in 86 (substituted for Ser in two), Tyr176 in all 88 (pseudo-conserved as Phe in five), Glu194 in 85 (substituted for Gln in two and for Arg in one) and Arg88 in 58 (substituted for Gln in 19 and for Lys in 10) of these orthologs (Supplementary Table S2). In addition to the information offered by the R88K and E194Q mutants about the specific contributions of the two residues to Zn^2+^ coordination in ZAC, the WT-like Zn^2+^ properties displayed by these mutants also demonstrate that Zn^2+^ functionality is likely to be retained in ZAC orthologs comprising Lys and Gln at positions 88 and 194, respectively. The fact that both charged and polar residues seem to be accommodated in these two positions in mammalian ZACs could suggest that these residues support Zn^2+^ coordination via hydrogen bonds, but they may also play a more structural role by contributing to the whole cation–π sphere. In light of this delineation of Zn^2+^ binding and the physicochemical similarities between zinc and the other transition metal ion ZAC agonist^17^, it is tempting to speculate that Cu^2+^ also targets and activates the receptor through this site, and spectral evidence does support the possibility of cation‒π interactions between Cu^2+^ and aromatic residues^34^. Collectively, these findings underscore the critical role of cation‒π interactions in ZAC function and broaden our understanding of the unique coordination sphere of zinc in different biological systems.

In addition to the delineation of the cation–π-based Zn^2+^ coordination to ZAC, the ZAC-Apo, ZAC-Zn_partial_ and ZAC-Zn structures also provide clues to some of the distinctive properties of the channel. The lack of loop C movement upon Zn^2+^ binding to ZAC and the similar conformations of the four Zn^2+^-coordinating residues in the ECD subunit interface in these structures (Supplementary Figs. S2a, S8) indicate the presence of a relatively weak preformed interaction network. We propose that this feature may explain the considerable levels of spontaneous activity exhibited by ZAC, reflecting an inherently low energy barrier between the resting and open channel states. In this scenario, the role of Zn^2+^ binding in ZAC activation would be to strengthen the interaction between the adjacent subunits forming this site without substantial rearrangement of the pocket, thereby further facilitating the conformational transition of the receptor from its resting state toward channel gating^17^. The further increased levels of spontaneous activity induced by the Y176W mutation could be interpreted to support the notion that it is the solidification of already existing interactions between the adjacent subunits that drives channel activation rather than any substantial movements of loops or regions in this site upon agonist binding. Collectively, these characteristics emphasize the importance of the intricate coordinating network involving aromatic, charged, and polar residues for efficient agonist binding and receptor activation.

Comparison of ZAC-Zn and ZAC^A152^-Zn revealed a putative intermolecular interaction network established at the narrowest distance between neighboring subunit ECDs that appears to be key for ZAC gating efficiency, judging from the dramatically smaller current amplitudes evoked by Zn^2+^ through homopentameric ZAC^A152^ than through homopentameric WT ZAC (Fig. 3d, f, g)^22^. Mutations of Leu126 appear to be almost as detrimental for Zn^2+^-evoked current amplitudes through ZAC as the Thr152Ala substitution (Fig. 3f, g), and the importance of the (−)-Thr152/(+)-Leu126 network for ZAC functionality is further supported by the complete conservation of the two residues across 88 mammalian nonhuman ZACs (Supplementary Table S2). The fact that a phenotype has yet to be reported for carriers of the Thr152Ala variant despite its remarkably high allelic frequency may reflect either that this attenuation of ZAC functionality does not produce an identifiable phenotype or that compensatory mechanisms are able to alleviate any variant-induced effects^22^. The high allelic frequency of this SNP makes the heterozygous ZAC/ZAC^A152^ combination the most prevalent genotype in almost all ethnicities, and — assuming similar transcription/translation efficiencies of the two alleles — these individuals will express a ZAC population of predominantly heteromeric ZAC/ZAC^A152^ assemblies characterized by a range of different WT/variant subunit stoichiometries and pentameric arrangements. The location of this interaction network at the ECD subunit interface thus raises the question of whether the Thr152Ala variant exerts a dominant negative effect manifested in the compromised functionality of heteromeric ZAC/ZAC^A152^ receptors or whether the presence of somewhere between one and four (−)-Thr152-comprising subunit interfaces in these heteromeric assemblies is sufficient to convey WT-like functionality. In any case, this identified network provides a plausible structural explanation for the pronounced consequences on ZAC gating arising from this SNP, and it also highlights a general activation mechanism wherein this and possibly other intermolecular networks facilitate the propagation of coordinated movements from the agonist binding site into interactions of the Cys loop (and a couple of other ECD loops) with the M2–M3 linker in the TMD, thus ultimately regulating channel pore dynamics.

While the intermolecular (−)-Thr152/(+)-Leu126 interaction thus appears to be a key structural element for transmitting agonist binding into ZAC gating, the intramolecular Cys289–Cys394 disulfide bond, by contrast, seems to act as a crucial brake on the conformational transition of ZAC from its resting state to its active state. Disruption of this bond via C289A and C394A mutations clearly shift the conformational equilibrium of ZAC toward the active state, resulting in pronounced ligand-independent channel activity (Fig. 2c). In their recent study, Lu et al. found that truncation of ZAC^A152^ after residue 392 (i.e., deletion of the entire C-terminus, including Cys394) resulted in constitutive activity, which they interpreted to support the proposed inhibitory effect of the C-terminus on ZAC function^21^. Interestingly, these characteristics of the ZAC^A152^(1–392) truncation mutant^21^ align with the high levels of constitutive activity displayed by C394A ZAC in this work (Fig. 2c), and the fact that C289A is highly constitutively active also suggests a key role for the disulfide bond in restricting ZAC in its resting state. Although our ZAC-Apo structure does not show the same wedging of the C-terminus into the ECD subunit interface as observed by Lu et al. in their ZAC^A152^-Apo structure^21^ (Fig. 2b), it is possible that the functional importance of the Cys289–Cys394 disulfide bond is rooted in its restriction of C-terminal flexibility, thereby enabling this docking interaction. Alternatively, the restrained structural flexibility of both the M2–M3 loop and the C-terminus produced by this disulfide bond could also influence the coupling efficiency between the ECD and TMD regions, ultimately leading to ZAC gating and thus contributing to the energy barrier favoring the resting state over the active state of ZAC. In any case, the complete conservation of both Thr152/Leu126 and Cys289/Cys394 across mammalian ZACs highlights how evolution has engineered inter- and intramolecular interactions into this receptor that govern its intrinsic activity and signaling properties while also tailoring them to either support or to be overcome by the conformational changes triggered by transmitter binding.

The delineation of the molecular mechanisms underlying the ZAC modulation exerted by two very different ZAC antagonists collectively demonstrates the conservation of some modulator binding sites across CLRs (*d*-TC) and identifies a novel location for a modulator site in a CLR (TTFB). In the case of *d*-TC, none of the numerous mutations introduced in our elaborate mutagenic explorations of *d*-TC sites 1 and 2 in ZAC led to substantial reductions in its antagonist potency at ZAC (Fig. 5d; Supplementary Figs. S5, S6, and Table S6). For site 1, the identified Zn^2+^-binding residues (Phe67, Tyr176, and Arg88) were observed to form cation–π or ionic interactions with the ammonium groups and aromatic rings of *d*-TC, and analogous cation–π interactions have been found to be pivotal for *d*-TC affinity to the corresponding site in nAChR^45^ (Fig. 5a; Supplementary Fig. S11). The site 1 residues investigated in this work may either form less important interactions with *d*-TC or merely line its binding pocket, in which case the impact of substitutions of these residues could be subtle. With respect to site 2, elimination of the side chains of three of the four residues observed to coordinate *d*-TC in the ZAC-TTFB structure does not substantially reduce its inhibitory potency. In light of the dual-action mechanism underlying *d*-TC-mediated ZAC inhibition, we cannot rule out that the failure to significantly impair *d*-TC binding to one of these two sites could be reflected in the WT-like inhibitory potency displayed by it in the tested mutants, including the sextuple R65L/N148L/Q214K/S277G/S278G/Q281G mutant (Fig. 5d; Supplementary Table S6). Alternatively, this failure to impact *d*-TC activity substantially through these mutations could suggest that its binding to ZAC may involve a more extensive or redundant network of interactions, where substitution of individual residues is insufficient to impact inhibition under our experimental conditions. Notably, the spatial orientations and binding modes of *d*-TC at its two sites in ZAC differ somewhat from those at the two corresponding *d*-TC sites in (α)_2_βγδ nAChR^45^, which could indicate some conformational flexibility of the antagonist at these sites that in turn could be at the root of the negligible effects produced by specific mutations. Consequently, the mutagenic data for *d*-TC presented here do not invalidate the structural observations but highlight a significant gap in our functional understanding.

Several of the pharmacophore elements identified in a previous small structure–activity relationship study of *N*-(thiazol-2-yl)-benzamide analogs as ZAC antagonists align well with the binding mode of TTFB to its binding site and the interactions formed by the inhibitor here^19^. For example, the beneficial effects of the presence of a 4-(tert-butyl)-substituent at the 1,3,4-thiadiazole ring (compared with both smaller and bulkier groups) and of a *meta-*halogen-substituent at the phenyl group for antagonist potency at ZAC^19^. TTFB targets a site in a ZAC region of obvious functional importance, and by being firmly wedged between adjacent M2 helices just above the 9’L residues, the inhibitor is likely to substantially restrict channel conformational dynamics. Unfortunately, the site location also complicated our mutagenic analysis, both by limiting which substitutions could be introduced without eliminating ZAC function and by the rather extreme channel properties exhibited by some of the functional mutants. Mutations of the 12’L, 13’V and 14’L residues forming the hydrophobic pocket for TTFB produced highly constitutively active channels such that the injected oocytes were of insufficient quality for recordings, and the introduction of bulky residues at positions 16’ or 17’ eliminated ZAC cell surface expression or function. While the ∼2-fold reduced antagonist potency displayed by TTFB in the S278A mutant could be indicative of the involvement of this 17’S residue in its binding, this subtle effect of the mutation could also be indirect (Fig. 4d; Supplementary Table S5). Overall, while the functional data presented here are insufficient to support the site observed in the ZAC-TTFB structure, the exploration of this site by mutagenesis is also so limited that the data do not invalidate this structural observation either. In contrast to *d*-TC, which, in addition to its promiscuous profile as a CLR antagonist, is a fairly complex molecule, TTFB, with its ZAC selectivity and simple *N*-(thiazol-2-yl)-benzamide scaffold, is much more suited as a lead for medicinal chemistry development. Thus, we propose that the atomic-scale insights into the binding site provided by the ZAC-TTFB structure provide a strong foundation for the structure-based design of new generations of TTFB analogs in the search for more potent and selective pharmacological tools for the receptor.

Finally, in addition to providing information about the structural basis for ZAC signaling and modulation, our findings provide important information for the identification of the putative physiological roles of the receptor. The identification of the ECD subunit interface as the region in ZAC harboring the binding site for Zn^2+^ (and likely also Cu^2+^) concords with the location of the canonical orthosteric sites in other CLRs. This is not as trivial a realization as it may seem, given the very different physicochemical properties of these metal ions compared with the small organic molecule agonists acting through the classical CLRs and the fact that Zn^2+^ and Cu^2+^ are known to act as modulators in many of these CLRs through a range of different allosteric sites^50–55^. In view of this, the ZAC activation elicited by Zn^2+^ binding to the ECD subunit interface strongly suggests that Zn^2+^ and Cu^2+^ could be orthosteric ZAC agonists. Both the composition of this site and other structural features in the ZAC pentamer, including the Thr152/Leu126 interaction network and the Cys289–Cys394 disulfide bond, will thus have been conserved via evolutionary pressure to maintain ZAC functionality in mammals. Intriguingly, the atypical cation–π-based coordination for Zn^2+^ observed in ZAC could be perceived as a consequence of the parallel evolution of ZAC and the classical CLRs from a common ancestral gene^15^, with the importance of this specific interaction for agonist binding to the orthosteric site being a shared heritage of these receptors. In any case, the structural and biochemical demonstration that ZAC is a mediator of Zn^2+^-elicited signaling could facilitate more focused explorations of the roles governed by the receptor in vivo. The apparent widespread expression of ZAC in the human body^14,15^ aligns well with the abundance of zinc in vivo, which further supports the role of the metal ion as an endogenous transmitter. The multitude of physiological processes regulated or contributed to by zinc still makes the search for putative ZAC functions a formidable task, which is further complicated by the absence of ZAC expression in the two most commonly used research animals (mice and rats). Nevertheless, this search can now be guided by evidence supporting its endogenous agonist.

## Materials and methods

### ZAC expression and purification

The full-length human *ZAC* was cloned and inserted into a BacMam vector modified with a GFP tag and a Strep-tag II at its C-terminus. Viral particles were generated in Sf9 cells, and 1 L of HEK293F cells at a density of 3 × 10⁶ cells/mL was transfected with 50 mL of P3 virus. The cells were cultured at 37 °C for 12 h, after which 10 mM sodium butyrate was added to increase protein expression. The culture was subsequently incubated at 30 °C for 48 h before the cells were harvested.

Cell pellets were resuspended in buffer A (50 mM Tris, pH 8.0, 150 mM NaCl) containing 1× protease inhibitor cocktail and were lysed mechanically using a Dounce tissue grinder. Membranes were extracted by adding 1% (w/v) DDM and 0.1% (w/v) CHS (both from Anatrace) and agitating the mixture at 4 °C for 3 h. After extraction, the supernatant was obtained via centrifugation at 12,000 rpm for 40 min at 4 °C and incubated with anti-strep affinity resin under gentle agitation at 4 °C for 3 h. The resin was collected using a gravity column, and the supernatant was incubated with fresh resin at 4 °C for an additional 3 h. The resin was then sequentially washed with decreasing concentrations of LMNG/CHS (1/0.1%; 0.1%/0.01%; 0.01%/0.001; final 0.001%/0.0001%) in buffer A. Finally, ZAC was eluted with 5 mM D-desthiobiotin in 0.001% LMNG and 0.0001% CHS buffer. Eluted ZAC solution was mixed with MSP1E3D1 and Brain Total Lipid (Avanti Polar Lipids) at a ratio of 1:7.5:200 and incubated at 4 °C for 40 min. Following incubation, Bio-Beads (Bio-Rad) were added to the mixture, which was gently agitated at 4 °C overnight. Fresh Bio-Beads were then added to the supernatant, and the mixture was agitated gently at 4 °C for an additional 2 h. The sample was subsequently centrifuged at 12,000 rpm for 10 min at 4 °C to remove the Bio-Beads and precipitate. The supernatant was subjected to a Superose6 10/300 GL column (GE Healthcare) in buffer containing 50 mM HEPES, 150 mM NaCl, and 50 μM ZnSO_4_ (pH 7.5). For the ZAC-TC or ZAC-TTFB samples, final 0.1 mM *d*-TC or TTFB solubilized in DMSO was mixed with the ZAC elution at a 10:1 molar ratio, followed by agitation at 4 °C for 30 min and size exclusion purification. Fractions were pooled for subsequent use.

### Cryo-EM sample preparation and data collection

Zn^2+^-bound ZAC^T152A^, *d-*TC-bound ZAC, and TTFB-bound ZAC samples were concentrated to ~3 mg/mL, whereas the Zn^2+^-bound WT ZAC sample was concentrated to ~5 mg/mL. In a separate experiment to achieve a higher Zn^2+^ concentration in the sample, additional ZnSO₄ was added to reach a final concentration of 250 μM. The mixture was centrifuged at 14,000 rpm for 5 min to remove any precipitates before freezing. Three hundred-mesh Au 1.2/1.3 grids (Quantifoil) were glow-discharged at 15 mA for 50 s using a PELCO easiGlo instrument. Then, 2.5 μL of the sample was applied to each grid, blotted for 4 s at 4 °C in 100% humidity using a vitrobot (FEI), and vitrified in liquid ethane cooled by liquid nitrogen. The frozen grids were stored under cryogenic conditions in liquid nitrogen for subsequent screening and cryo-EM data collection.

All datasets were collected on a Titan Krios G4 cryo-electron microscope operated at 300 kV and equipped with a Falcon G4i direct electron detector and a Selectris X imaging filter (Thermo Fisher Scientific) with a 20 eV slit size. Movie stacks were acquired in super-resolution mode using EPU software (Thermo Fisher Scientific) with a defocus of −2.0 μm and a final calibrated pixel size of 0.932 Å. Each electron event representation (EER) movie represented a total dose of 50 e⁻/Å².

### Image processing

For the ZAC-Apo sample, an initial dataset of 2744 EER movies was collected using a Falcon G4i detector and fractionated into 40 subframes. Beam-induced motion correction was performed with a MotionCor2-like algorithm implemented in RELION (v3.1)^56^. The exposure-weighted micrographs were imported into cryoSPARC (v3.3.2)^57^, where contrast transfer function (CTF) estimation was carried out using CTFFIND4^58^. A total of 3,364,832 particles were retained after the results of blob-picking were inspected and extracted with a box size of 220 pixels (binned by 3×). A subset of particles underwent 2D classification, and 103,631 high-quality particles were selected. These particles were converted for re-extraction in RELION, followed by auto-refinement and Bayesian polishing. The 16,419 polished particles were imported back into cryoSPARC for multiple rounds of heterogeneous refinement. The final 3.35 Å map for apo ZAC was reconstructed via non-uniform refinement with C1 symmetry. Map resolution was estimated within cryoSPARC using the gold-standard Fourier shell correlation (FSC) at the 0.143 criterion.

For the Zn^2+^-ZAC sample treated with 50 μM Zn^2+^ in nanodiscs, an initial dataset of 4539 EER movies was collected using a Falcon G4i detector and fractionated into 40 subframes. Beam-induced motion correction was performed with a MotionCor2-like algorithm implemented in RELION (v3.1)^56^. The exposure-weighted micrographs were imported into cryoSPARC (v3.3.2)^57^, where CTF estimation was carried out using CTFFIND4^58^. A total of 6,688,609 particles were retained after the results of blob-picking were inspected and extracted with a box size of 220 pixels (binned by 3×). A subset of particles underwent 2D classification, and 374,247 high-quality particles were selected. These particles were converted for re-extraction in RELION, followed by auto-refinement and Bayesian polishing. The polished particles were imported back into cryoSPARC for ab initio reconstruction and multiple rounds of heterogeneous refinement, resulting in the isolation of an apo ZAC map and a Zn^2+^-partially occupied ZAC map. The particles were further optimized with global and local CTF refinement. The final 3.03 Å map for apo ZAC was reconstructed using 17,577 particles via homogeneous refinement with C5 symmetry. For the Zn²⁺ partially occupied ZAC, the final 2.85 Å map was reconstructed using 111,743 particles with local refinement at C1 symmetry. Map resolution was estimated within cryoSPARC using the gold-standard FSC at the 0.143 criterion.

Datasets of WT ZAC treated with 250 μM Zn^2+^ (10,650 EER), TC (2263 EER), TTFB (4311 EER), and ZAC^T152A^-Zn^2+^ (9002 EER) were processed following a similar workflow. Polished particles from RELION were imported back into cryoSPARC for ab initio reconstruction and multiple rounds of heterogeneous refinement. Symmetry expansion with C5 symmetry, 3D variability analysis, and global and local CTF refinement were applied for further optimization.

### Model building

The initial ZAC model was obtained from the AlphaFold database^59^. This predicted model was rigid-body docked into the ZAC cryo-EM density map using ChimeraX (v1.6)^60^. Subsequent iterative manual adjustments were performed in COOT (v0.9.8)^61^, followed by real-space refinement using Phenix (v1.19)^62^. Model validation was carried out using MolProbity^63^, and sidechains lacking well-defined density were trimmed before deposition. The final refinement statistics are summarized in Supplementary Table S1. Structural figures were generated using ChimeraX or PyMOL (PyMOL Molecular Graphics System, v2.3.4, Schrödinger) (https://pymol.org/2/).

### Molecular biology for functional studies

All WT and mutant *ZAC* cDNAs used for electrophysiology studies were in the pUNIV vector (Addgene, Watertown, MA), and the construction of the WT ZAC-pUNIV plasmid has been reported previously^22^. ZAC mutant cDNAs were generated using a QuikChange mutagenesis kit (Stratagene, Santa Clara, CA) or the overlap extension PCR technique^64^ applying oligonucleotides from TAG Copenhagen A/S (Copenhagen, Denmark). The integrity and absence of unwanted mutations in all cDNAs created by PCR were verified by DNA sequencing (Macrogen Europe, Amsterdam, The Netherlands). For cRNA generation, cDNAs were linearized using the restriction enzyme NotI (New England Biolabs, Ipswich, MA) and subsequently transcribed and capped using the mMessage mMachine T7 RNA Transcription Kit (Ambion, Waltham, MA).

### *Xenopus* oocyte injections and TEVC recordings

Defolliculated stage V-VI oocytes harvested from female *Xenopus laevis* frogs were obtained from Lohmann Research Equipment (Castrop-Rauxel, Germany). Unless otherwise indicated, the oocytes were injected with 9.2, 18.4, or 36.8 nL of cRNA solution at a concentration of 100 ng/μL cRNA, i.e., 0.92, 1.8,4 or 3.68 ng of cRNA, respectively. In those cases where oocytes injected with these cRNA amounts for a specific ZAC mutant did not exhibit significant or only exhibited negligible current responses to Zn^2+^ application, oocytes were injected with higher amounts of cRNAs in the following rounds (40–70 ng cRNA per oocyte). For all the mutants injected in these high cRNA quantities that were found to be non-responsive to Zn^2+^, another cRNA sample was prepared, injected into the oocytes, and tested. Following cRNA injection, the oocytes were incubated in sterile modified Barth’s solution (88 mM NaCl, 1 mM KCl, 15 mM HEPES (pH 7.5), 2.4 mM NaHCO_3_, 0.41 mM CaCl_2_, 0.82 mM MgSO_4_, 0.3 mM Ca(NO_3_)_2_, 100 U/mL penicillin and 100 μg/mL streptomycin) at 16–18 °C. Oocytes injected with cRNAs encoding ZAC mutants characterized by high levels of constitutive activity were incubated in sterile modified Barth’s solution supplemented with *d-*TC (100 μM). All TEVC recordings were performed two or three days after cRNA injection.

On the day of the TEVC recordings, all compound dilutions were prepared in a saline solution (115 mM NaCl, 2.5 mM KCl, 10 mM MOPS, 1.8 mM CaCl_2_, 0.1 mM MgCl_2_, pH 7.5). Oocytes were placed in a recording chamber continuously perfused with this saline solution, and the compounds were applied to the perfusate. Both the voltage and current electrodes were agar-plugged with 3 M KCl with a resistance of 0.2–2.0 MΩ. Oocytes were voltage-clamped at −50 mV by a Gene Clamp 500B amplifier, and current signals were digitized by a Digidata 1322 A (both from Axon Instruments, Union City, CA). Currents were recorded using pCLAMP 10 (Molecular Devices, Sunnyvale, CA). The recordings were performed at room temperature.

In all recordings, the compound or compound combinations were applied in the bath until the peak current decayed to a steady state or for up to 30 s. As also observed in previous studies of ZAC^22^, the currents evoked by sub-saturating concentrations of Zn^2+^ at ZAC did not reach well-defined peaks during the 30-s application, but the pharmacological properties displayed by the agonists at the receptor were nevertheless reflected well by the data extracted from these recordings. At the beginning of all recordings, two consecutive applications of the same Zn^2+^ concentration were applied to the oocyte, and it was verified that these consecutive applications elicited responses of comparable current amplitudes ( ± 20%). For oocytes used for Zn^2+^ concentration–response relationship determinations, Zn^2+^ (10 mM) giving rise to the I_max_ was used, and for oocytes used for *d*-TC and TTFB concentration-inhibition relationship determinations, Zn^2+^ (1 mM) was used. The antagonist properties of *d*-TC and TTFB were determined by pre-application of the compound to the perfusate for 30 s followed by co-application of the compound and Zn^2+^ (1 mM). In all recordings, washes of 30–60 s were executed between the ligand applications.

### TEVC data analysis

Data analysis was performed using Clampfit software version 10.5 (Molecular Devices, Crawley, UK) and GraphPad Prism version 10.4.1 (GraphPad Software, La Jolla, CA). For Zn^2+^ concentration–response relationship data, the inward currents induced by different Zn^2+^ concentrations in WT and mutant ZAC-expressing oocytes were normalized to the Zn^2+^ I_max_ from the fitted concentration‒response curve. For *d*-TC and TTFB concentration–inhibition relationship data, the positive (upward) changes in holding currents induced by the antagonist during preincubation were normalized to the positive (upward) change in holding current mediated by 100 μM antagonist (I_100__μM_ _*d*-TC_ or I_100__μM TTFB_), and the reduced inward currents induced by Zn^2+^ (1 mM) in the presence of various antagonist concentrations were normalized to the response elicited by Zn^2+^ (1 mM) alone (I_1__mM_ _Zn_^2+^) in the specific oocyte. Concentration‒response and concentration‒inhibition curves were fitted in GraphPad Prism by nonlinear regression using the equation for sigmoidal dose‒response with variable slope. Each data point represents the mean ± SEM of recordings performed on at least five oocytes in total (*n*) from at least two different batches.

## Supplementary information

Supplementary Information

## Data Availability

The cryo-EM maps have been deposited into the Electron Microscopy Data Bank (EMDB) under accession numbers: EMBD-63035 for ZAC-Apo, EMBD-63033 for ZAC-Zn, EMBD-63034 for ZAC^A152^-Zn, EMBD-63036 for ZAC-Zn_partial_, EMBD-63037 for ZAC-*d*-TC, EMBD-63038 for ZAC-TTFB. The coordinates have been deposited at the Protein Data Bank (PDB) under accession numbers: 9LEV for ZAC-Apo, 9LET for ZAC-Zn, 9LEU for ZAC^A152^-Zn, 9LEX for ZAC-Zn_partial_, 9LEY for ZAC-*d*-TC, 9LEZ for ZAC-TTFB.
