# Supplementary material for: Structural basis of human zinc-activated channel (ZAC) signaling and modulation
Source: Cell Discov. 2026 Mar 31;12:23. doi: 10.1038/s41421-026-00878-5 (PMC13036004; doi:10.1038/s41421-026-00878-5)
Supplement: Supplementary file 1 — Supplementary Information [file 41421_2026_878_MOESM1_ESM.pdf]

Structural basis of human zinc-activated channel (ZAC) signaling and modulation

Zixuan Zhou[1], Yonghui Long[1], Yulin Chao[1], Chuanhui Yang[1], Yi-Quan Tang[5], Yilai Shu[4], Hongtao Zhu[3*], Anders A. Jensen[2*], Qianhui Qu[1*]

[1]Eye & ENT Hospital, Institutes of Biomedical Sciences，Shanghai Key Laboratory of Medical Epigenetics, International Co-laboratory of Medical Epigenetics and Metabolism (Ministry of Science and Technology), Department of Systems Biology for Medicine, Fudan University, Shanghai, China.

[2]Department of Drug Design and Pharmacology, Faculty of Health and Medical Sciences, University of Copenhagen, Copenhagen, Denmark

[3]Beijing National Laboratory for Condensed Matter Physics, Institute of Physics, Chinese Academy of Sciences, Beijing, China

[4]ENT Institute and Otorhinolaryngology Department of Eye & ENT Hospital, Fudan University, Shanghai, China

[5]State Key Laboratory of Medical Neurobiology and MOE Frontiers Center for Brain Science, Institutes of Brain Science, Fudan University, Shanghai, China

Correspondence: qqh@fudan.edu.cn; aaj@sund.ku.dk; hongtao.zhu@iphy.ac.cn

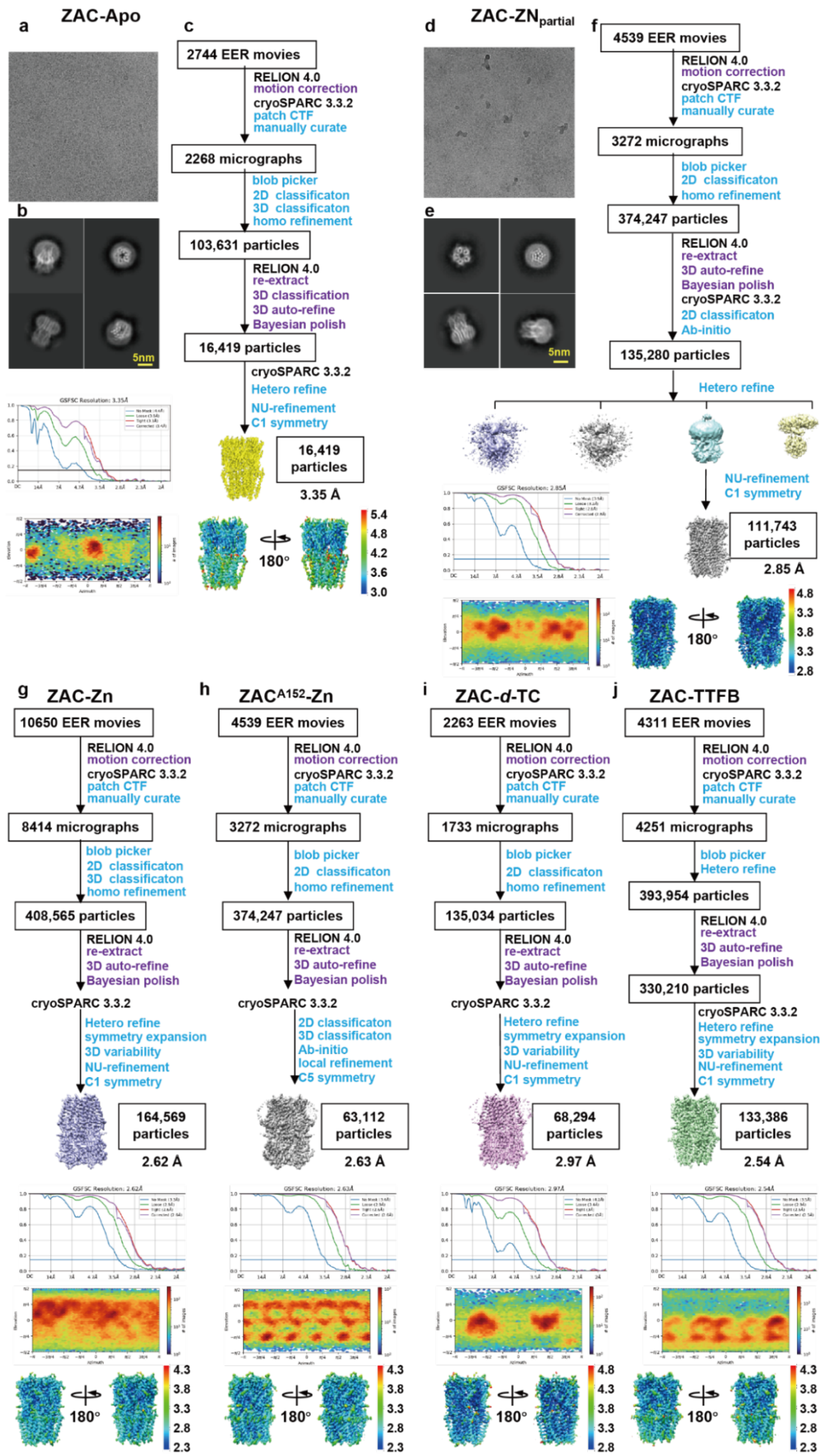

a
ZAC-Apo
b
5nm
c
2744 EER movies
RELION 4.0
motion correction
cryoSPARC 3.3.2
patch CTF
manually curate
2268 micrographs
blob picker
2D classificaton
3D classificaton
homo refinement
103,631 particles
RELION 4.0
re-extract
3D classification
3D auto-refine
Bayesian polish
16,419 particles
cryoSPARC 3.3.2
Hetero refine
NU-refinement
C1 symmetry
16,419 particles
3.35 Å
180°
d
ZAC-ZNpartial
e
5nm
f
4539 EER movies
RELION 4.0
motion correction
cryoSPARC 3.3.2
patch CTF
manually curate
3272 micrographs
blob picker
2D classificaton
homo refinement
374,247 particles
RELION 4.0
re-extract
3D auto-refine
Bayesian polish
cryoSPARC 3.3.2
2D classificaton
Ab-initio
135,280 particles
Hetero refine
NU-refinement
C1 symmetry
111,743 particles
2.85 Å
180°
g
ZAC-Zn
10650 EER movies
RELION 4.0
motion correction
cryoSPARC 3.3.2
patch CTF
manually curate
8414 micrographs
blob picker
2D classificaton
3D classificaton
homo refinement
408,565 particles
RELION 4.0
re-extract
3D auto-refine
Bayesian polish
cryoSPARC 3.3.2
Hetero refine
symmetry expansion
3D variability
NU-refinement
C1 symmetry
164,569 particles
2.62 Å
180°
h
ZACA152-Zn
4539 EER movies
RELION 4.0
motion correction
cryoSPARC 3.3.2
patch CTF
manually curate
3272 micrographs
blob picker
2D classificaton
homo refinement
374,247 particles
RELION 4.0
re-extract
3D auto-refine
Bayesian polish
cryoSPARC 3.3.2
2D classificaton
3D classificaton
Ab-initio
local refinement
C5 symmetry
63,112 particles
2.63 Å
180°
i
ZAC-d-TC
2263 EER movies
RELION 4.0
motion correction
cryoSPARC 3.3.2
patch CTF
manually curate
1733 micrographs
blob picker
2D classificaton
homo refinement
135,034 particles
RELION 4.0
re-extract
3D auto-refine
Bayesian polish
cryoSPARC 3.3.2
Hetero refine
symmetry expansion
3D variability
NU-refinement
C1 symmetry
68,294 particles
2.97 Å
180°
j
ZAC-TTFB
4311 EER movies
RELION 4.0
motion correction
cryoSPARC 3.3.2
patch CTF
manually curate
4251 micrographs
blob picker
Hetero refine
393,954 particles
RELION 4.0
re-extract
3D auto-refine
Bayesian polish
330,210 particles
cryoSPARC 3.3.2
Hetero refine
symmetry expansion
3D variability
NU-refinement
C1 symmetry
133,386 particles
2.54 Å
180°

Supplementary Fig. S1. Cryo-EM analysis of ZAC with different ligands.

a, Representative raw image of ZAC-Apo. **b,** Representative 2D classification results of ZAC-Apo. **c,** Processing workflow for ZAC protein in apo state. **d,** Representative raw image of ZAC in zinc-partially bound state. **e,** Representative 2D classification results of ZAC in zinc-partially bound state. **f-j,** Processing workflow for ZAC protein in zinc-partially bound state (**f**), zinc-fully bound state (**g**), zinc-fully bound ZAC^{A152} state (**h**), *d*-TC-bound state (**i**) and TTFB-bound state (**j**). For each state, the map resolution was estimated using the gold-standard Fourier shell correlation (GSFSC) with a cutoff at 0.143. The angular distribution heatmap for the final particles used in the reconstructions is shown. The final reconstructed maps are color-coded by local resolution.

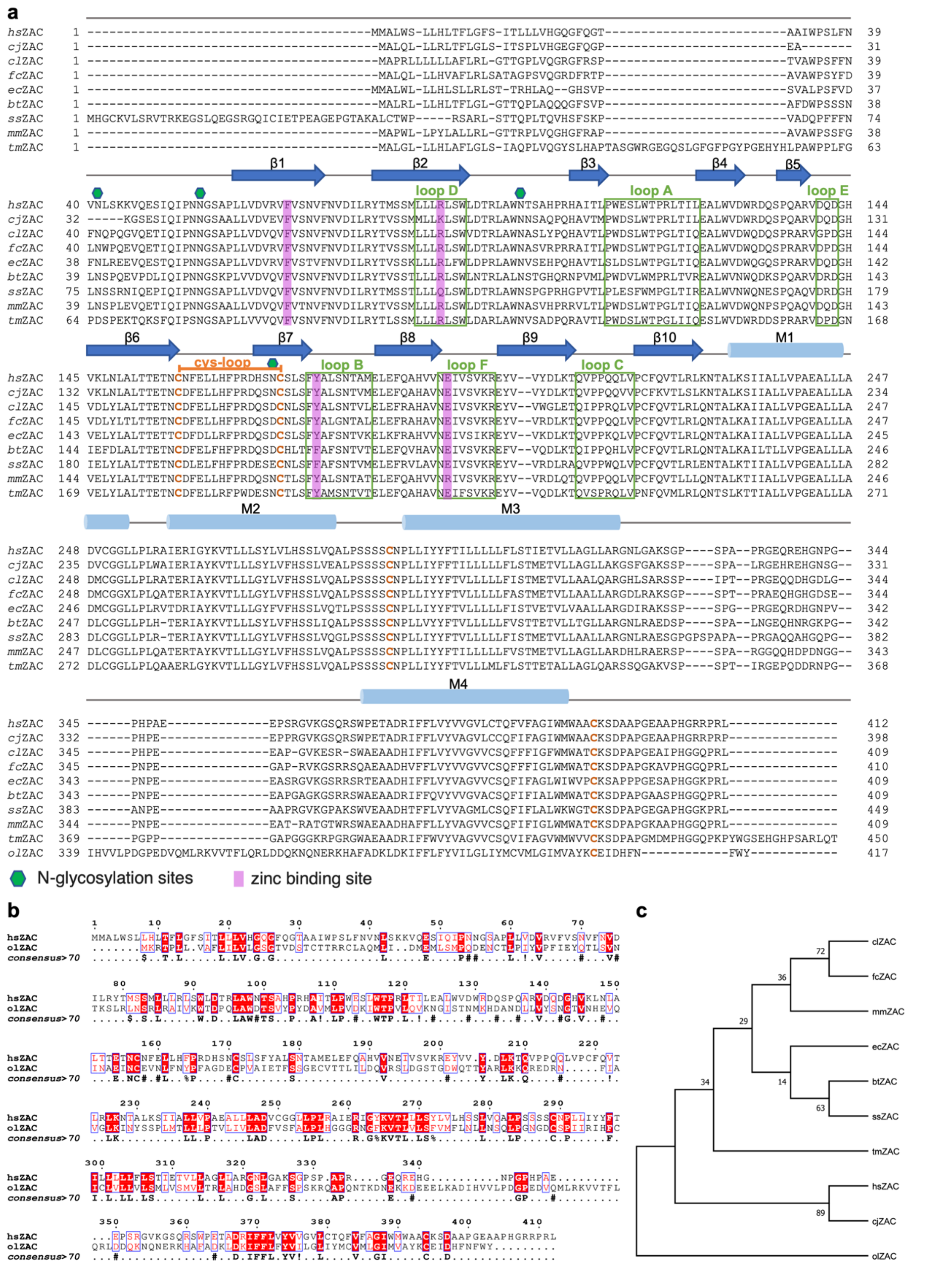

Supplementary Fig. S2. Sequence alignment and conservation analyses of ZAC proteins in different species.

a, The sequence of ZAC homologs from human (*hs*ZAC, Accession Number NM_180990), marmoset (*cj*ZAC, Accession Number XM_008997774), dog (*cf*ZAC, Accession Number NM_001010955), cat (*fc*ZAC, Accession Number XM_011289384.4), horse (*ec*ZAC, Accession Number XM_014737539.3), cow (*bt*ZAC, Accession Number NM_001191252), pig (*ss*ZAC, Accession Number XM_021066603), bat (*mm*ZAC, Accession Number XM_036269489.1),

manatee (*tm*ZAC, Accession Number XM_004374386). The secondary structural segments of ZAC are shown above in reference to the human ZAC structure. Key features such as residues involved in zinc binding are highlighted in magenta, N-glycosylation sites in green hexagons, and residues contributing to disulfide bonds in orange. **b,** Comparison of human ZAC and putatively *Oryzias latipes* (*ol*ZAC, GI: 2809435460), using ESPript 3.2 online server. Residues with consensus scores greater than 70 are highlighted below the alignment results. **c,** Evolutionary relationships of mammalian ZAC homologs and olZAC. The evolutionary history was inferred using the Neighbor-Joining method and the evolutionary analyses were conducted in MEGA11.

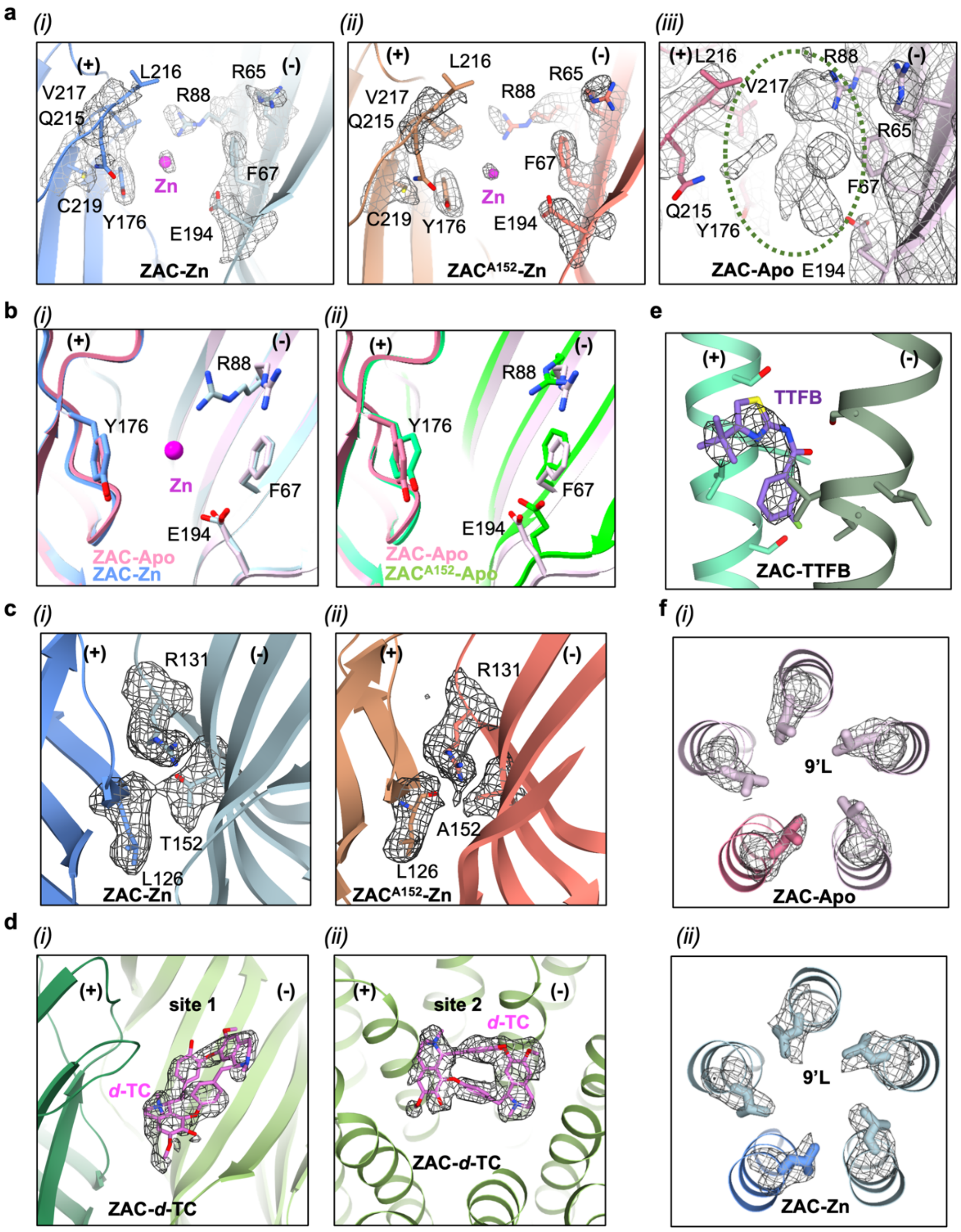

Supplementary Fig. S3. Density maps of zinc and antagonists.

a, The density maps of the Zn^{2+}-binding site at the ECD interface of ZAC-Zn (***i***), ZAC^{A152}-Zn (***ii***) and ZAC-Apo states (***iii***) are shown, with density contoured at 4.5 σ. Surrounding residues are shown in sticks for comparison. **b,** Structural alignment between ZAC-Apo and ZAC-Zn ***(i)*** or ZAC^{A152}-Apo (***ii***, PDB 8YX8). **c,** The density maps of (+)-L126, (-)-R131 and (-)-T152 or (-)-A152 in the ZAC-Zn ***(i)*** and ZAC^{A152}-Zn ***(ii)*** structures are shown at 1.7 σ. **d,** The density map of *d*-TC in ZAC-*d*-TC is shown at 1.80 σ, with *d*-TC depicted in orchid sticks. **e,** The density map for TTFB in ZAC-TTFB is shown at 2.96 σ, with TTFB depicted in purple sticks. **f,** The density maps of 9'L in ZAC-Apo ***(i)*** and ZAC-Zn ***(ii)***. For all these structures, the principal subunit interface (+) and the complementary subunit interface (-) are color-coded differently for clarity.

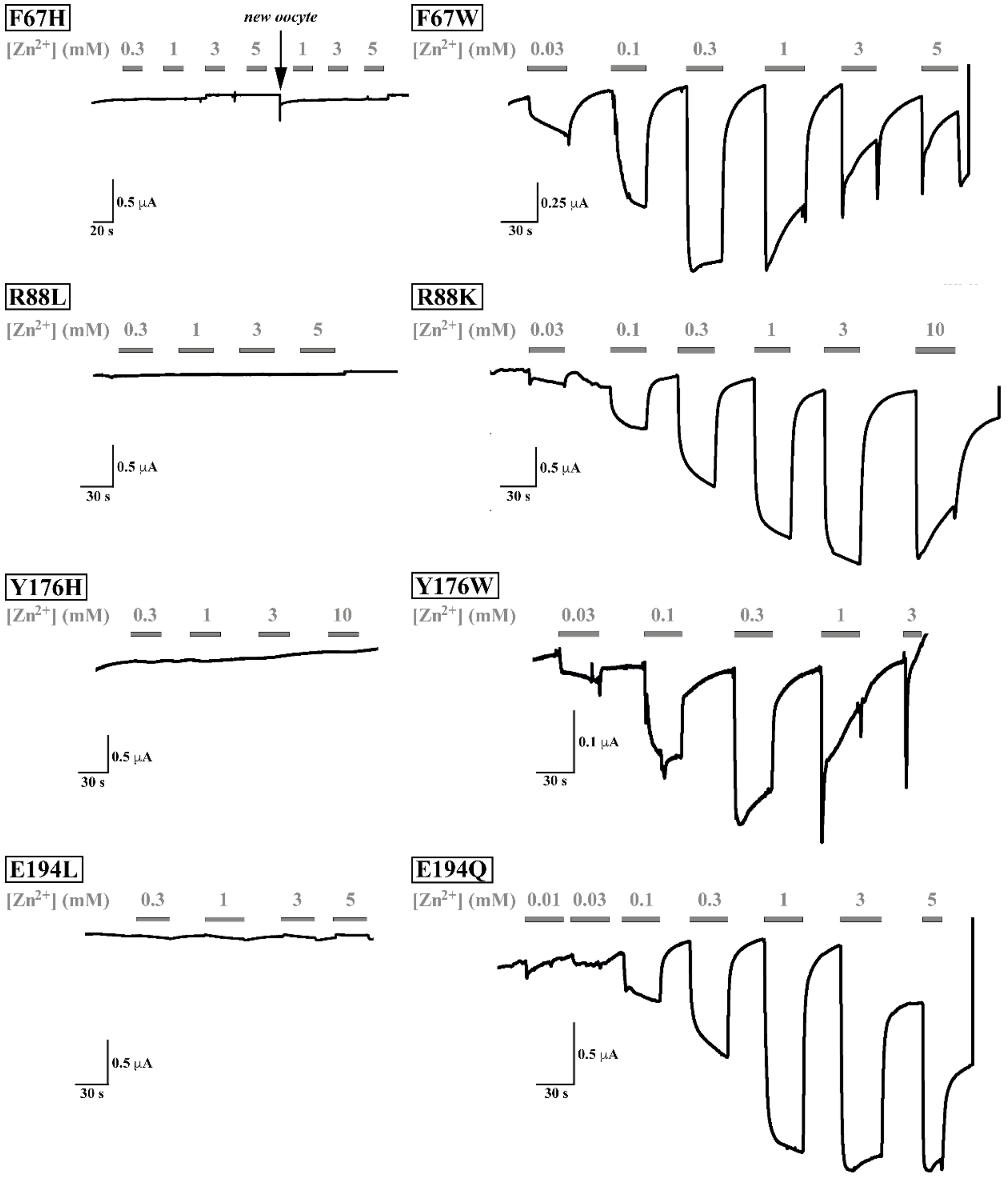

Supplementary Fig. S4. Functional properties of Zn^{2+} at zinc-binding site ZAC mutants. Representative traces of the current responses evoked by Zn^{2+} in *Xenopus* oocytes expressing 8 zinc-binding site ZAC mutants (the mutants not shown in Fig. 1e): F67H, F67W, R88L, R88K, Y176H, Y176W, E194L and E194Q.

a

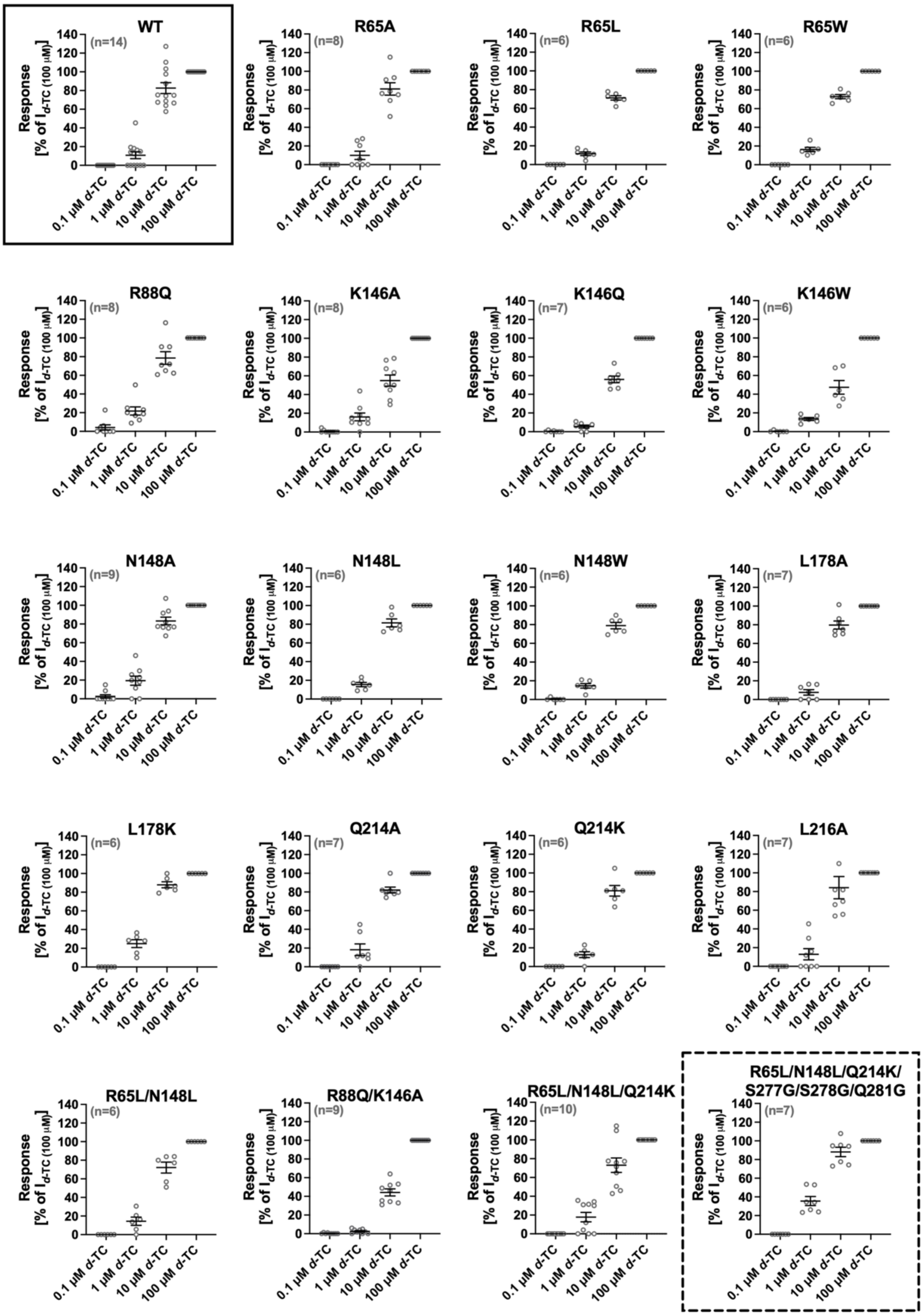
WT
(n=14)
R65A
(n=8)
R65L
(n=6)
R65W
(n=6)
R88Q
(n=8)
K146A
(n=8)
K146Q
(n=7)
K146W
(n=6)
N148A
(n=9)
N148L
(n=6)
N148W
(n=6)
L178A
(n=7)
L178K
(n=6)
Q214A
(n=7)
Q214K
(n=6)
L216A
(n=7)
R65L/N148L
(n=6)
R88Q/K146A
(n=9)
R65L/N148L/Q214K
(n=10)
R65L/N148L/Q214K/S277G/S278G/Q281G
(n=7)
Response [% of I d-TC (100 μM)]
0.1 μM d-TC
1 μM d-TC
10 μM d-TC
100 μM d-TC

b

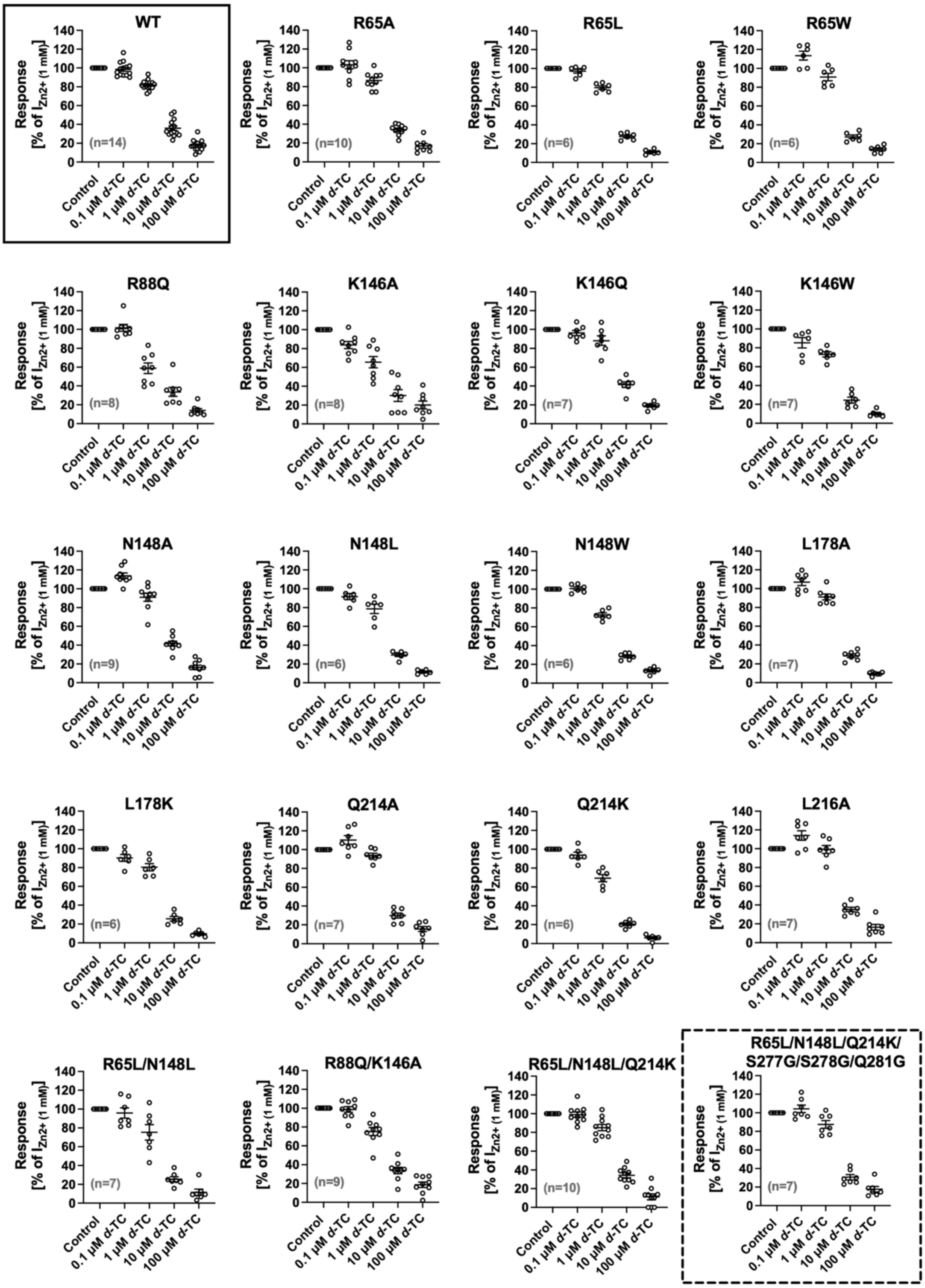

Supplementary Fig. S5. Antagonist properties displayed by *d*-TC at the *d*-TC site 1 ZAC mutants.

a. *d*-TC-mediated antagonism of the spontaneous activity of wild-type (WT) ZAC and *d*-TC site 1 mutants expressed in *Xenopus* oocytes in TEVC recordings. Data for the inhibition of the

spontaneous activity represent positive (upward) changes in holding currents produced by *d*-TC during the 30-s preincubation and are normalized to the positive (upward) change in holding currents produced by *d*-TC (100 μM) [$I_{d\text{-TC}\ (100\ \mu M)}$] at the oocytes. **b.** *d*-TC-mediated antagonism of the Zn^{2+} (1 mM)-evoked response though wild-type ZAC and *d*-TC site 1 mutants expressed in *Xenopus* oocytes in TEVC recordings. Data for the inhibition of the Zn^{2+} (1 mM)-evoked response represent the reduced inward currents produced by subsequent co-application of Zn^{2+} (1 mM) and *d*-TC and are normalized to the current produced by Zn^{2+} (1 mM) alone [$I_{Zn2+\ (1\ mM)}$] at the oocytes. The fitted IC_{50} values for *d*-TC as antagonist of the Zn^{2+}-evoked responses though WT and mutant ZAC are given in Supplementary Table S3. (**a, b**) Data are given as the individual data points with mean ± S.E.M. values. Data for WT ZAC and for the *d*-TC sites 1+2 mutant ZAC-R65L/N148L/Q214K/S277G/S278G/Q281G are indicated with solid and hatches boxes, respectively.

a

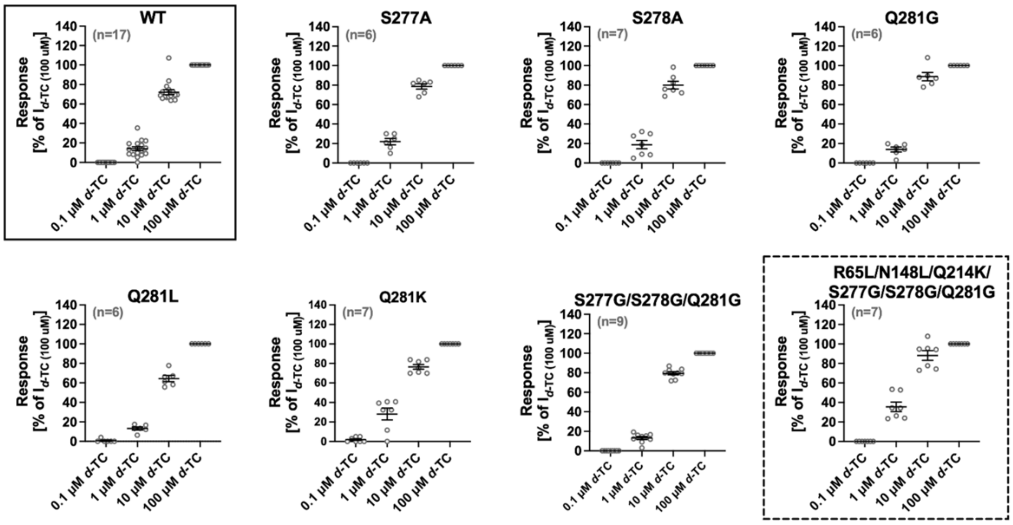

b

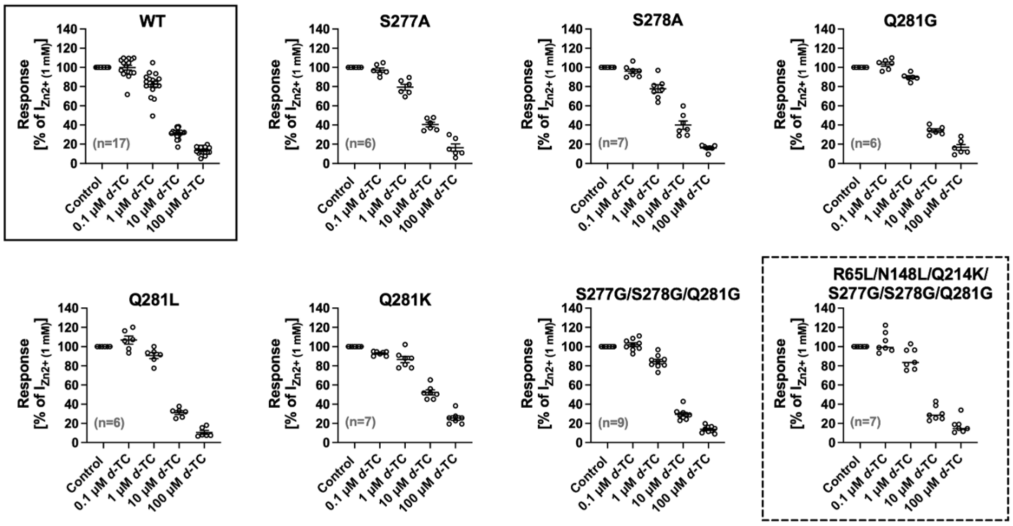

Supplementary Fig. S6. Antagonist properties displayed by *d*-TC at the *d*-TC site 2 ZAC mutants.

a. *d*-TC-mediated antagonism of the spontaneous activity of wild-type (WT) ZAC and *d*-TC site 2 mutants expressed in *Xenopus* oocytes in TEVC recordings. Data for the inhibition of the spontaneous activity represent positive (upward) changes in holding currents produced by *d*-TC during the 30-s preincubation and are normalized to the positive (upward) change in holding currents produced by *d*-TC (100 μM) [$I_{d\text{-TC (100 μM)}}$] at the oocytes. **b.** *d*-TC-mediated antagonism of the Zn^{2+} (1 mM)-evoked response though wild-type ZAC and *d*-TC site 2 mutants expressed in *Xenopus* oocytes in TEVC recordings. Data for the inhibition of the Zn^{2+} (1 mM)-evoked response represent the reduced inward currents produced by subsequent co-application of Zn^{2+} (1 mM) and *d*-TC and are normalized to the current produced by Zn^{2+} (1 mM) alone [$I_{Zn2+\text{ (1 mM)}}$] at the oocytes. The fitted IC_{50} values for *d*-TC as antagonist of the Zn^{2+}-evoked responses though WT and mutant ZAC are given in Supplementary Table S6. Data are given as the individual data points with mean ± S.E.M. values. Data for WT ZAC and for the *d*-TC sites 1+2 mutant ZAC-R65L/ N148L/Q214K/S277G/S278G/Q281G are indicated with solid and hatches boxes, respectively.

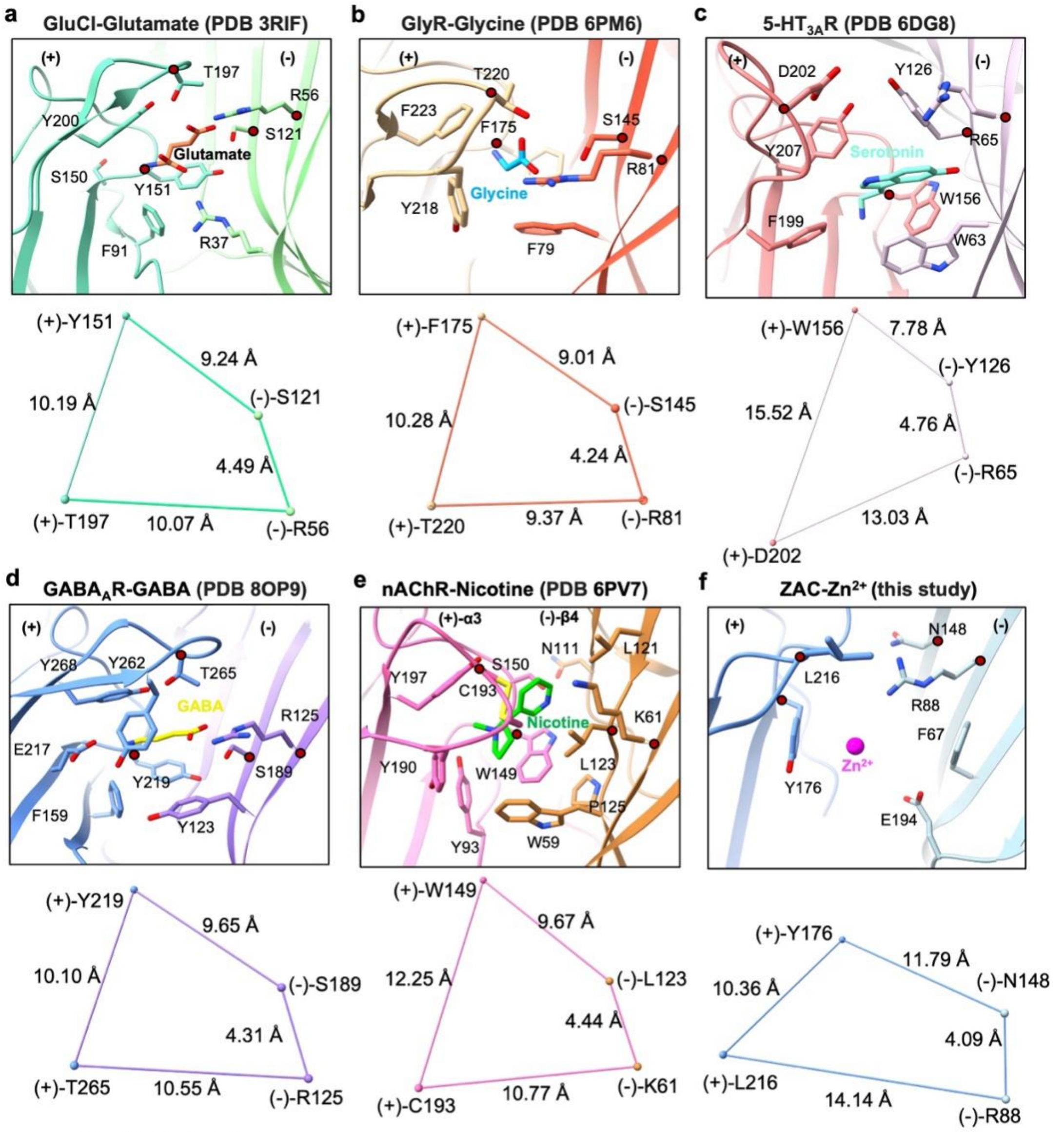

Supplementary Fig. S7. The canonical orthosteric sites at the ECD subunit interface of CLRs.

Detailed structural information of **a,** the glutamate binding site in GluCl (PDB 3RIF). **b,** the glycine binding site in α1 GlyR (PDB 6UD3). **c,** the serotonin binding site in 5-HT_{3A}R (PDB 6DG8). **d,** the GABA binding site in ρ1 $GABA_A$R (PDB 8OP9). **e,** the nicotine binding site in α3β4 nAChR (PDB 6PV7). **f,** the zinc binding site in human ZAC (this study). The sizes of agonist binding pockets are illustrated by the Cα atom distances of key residues shown below respectively. The principal subunit interface (+) and the complementary subunit interface (-) are colored differently.

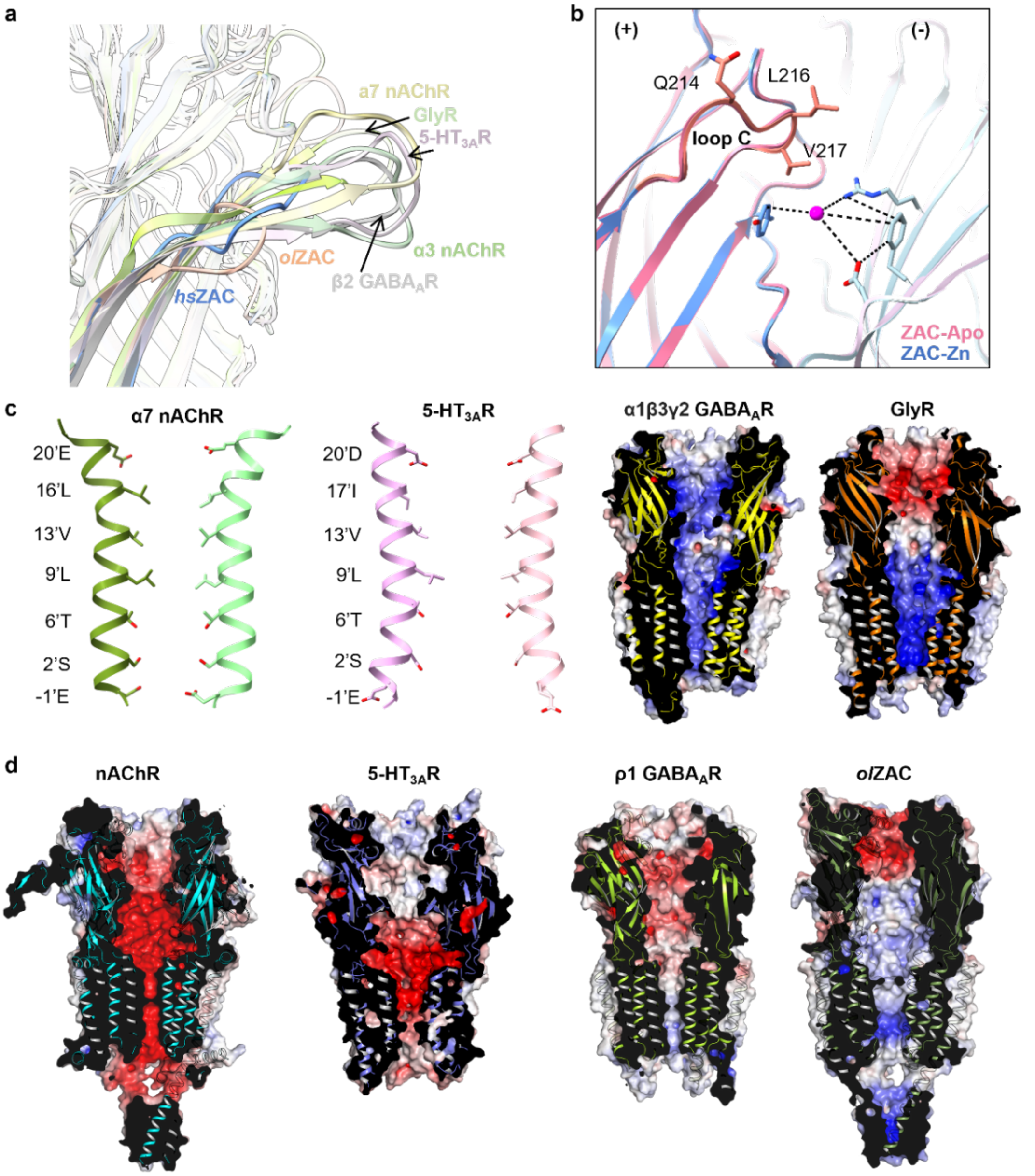

Supplementary Fig. S8. Loop C and ion permeation pathways in CLRs.

a, Structural comparison of loop C in *hs*ZAC (blue), *ol*ZAC (PDB 8WGE, orange), β2 $GABA_AR$ (PDB 6D6T, gray), α3 nAChR (PDB 6PV7, green), α7 nAChR (PDB 8V80, light yellow), 5-$HT_{3A}R$ (PDB 6DG8, thistle) and α1 GlyR (PDB 6UD3, light green). Secondary structures, except loop C, are shown in transparency. **b,** Detailed structural landscape of loop C near the zinc-binding site in human ZAC-Zn and ZAC-Apo. Mutated residues on loop C, which were assessed for their impact on zinc response (Supplementary Fig. S5), are highlighted in sticks. **c,** The channel pore of cation channels α7 nAchR and 5-$HT_{3A}R$ depicted through two M2 helices. The residues lining the permeation pathway are shown as sticks. The principal subunit interface (+) and the complementary subunit interface (-) are colored differently. **d,** The ion permeation pathway of CLRs. Solvent-accessible electrostatic potential mapped on the receptor surface of α7 nAChR, 5-HT_3AR, *ol*ZAC (PDB 8WGE), ρ1 $GABA_AR$ (PDB 8OQ6), α1β3γ2 $GABA_AR$ (PDB 6I53) and α1 GlyR (PDB 3JAF), color-coded from -10 kT^{e-1} to +10 kT^{e-1} (red to blue).

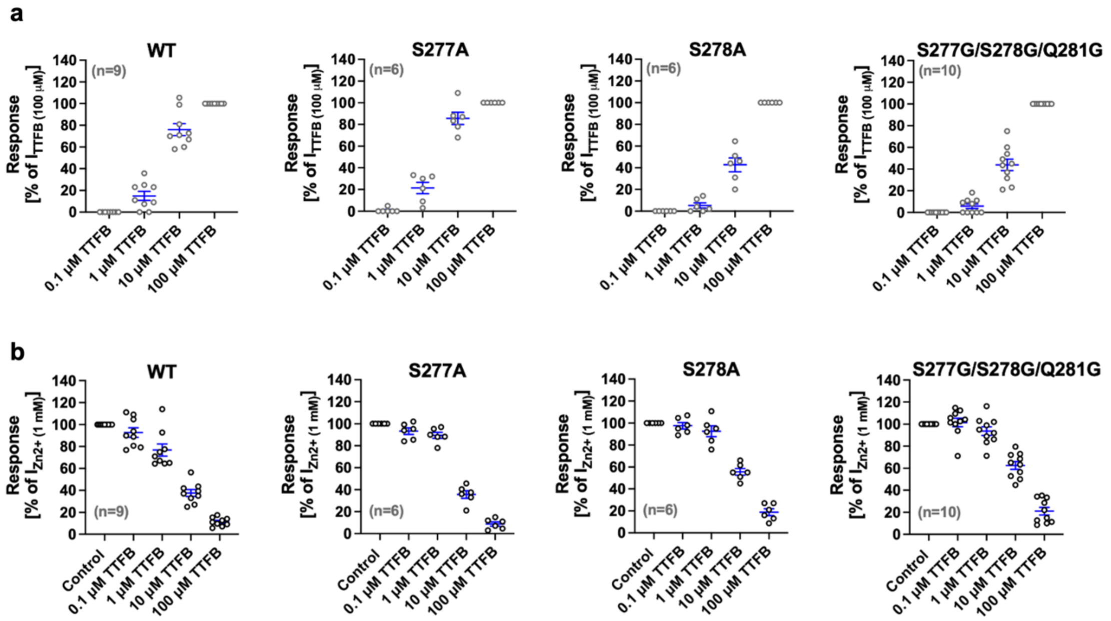

Supplementary Fig. S9. Antagonist properties displayed by TTFB.

a. TTFB-mediated antagonism of the spontaneous activity of wild-type (WT) ZAC and mutants expressed in *Xenopus* oocytes in TEVC recordings. Data for the inhibition of the spontaneous activity represent positive (upward) changes in holding currents produced by TTFB during the 30-s preincubation and are normalized to the positive (upward) change in holding currents produced by TTFB (100 μM) [$I_{TTFB\ (100\ \mu M)}$] at the oocytes. **b,** TTFB-mediated antagonism of the Zn^{2+} (1 mM)-evoked response though wild-type ZAC and mutants expressed in *Xenopus* oocytes in TEVC recordings. Data for the inhibition of the Zn^{2+} (1 mM)-evoked response represent the reduced inward currents produced by subsequent co-application of Zn^{2+} (1 mM) and TTFB and are normalized to the current produced by Zn^{2+} (1 mM) alone [$I_{Zn2+\ (1\ mM)}$] at the oocytes. The fitted IC_{50} values for TTFB as antagonist of the Zn^{2+}-evoked responses though WT and mutant ZAC are given in Supplementary Table S5. (**a, b**) Data are given as the individual data points with mean ± S.E.M. values.

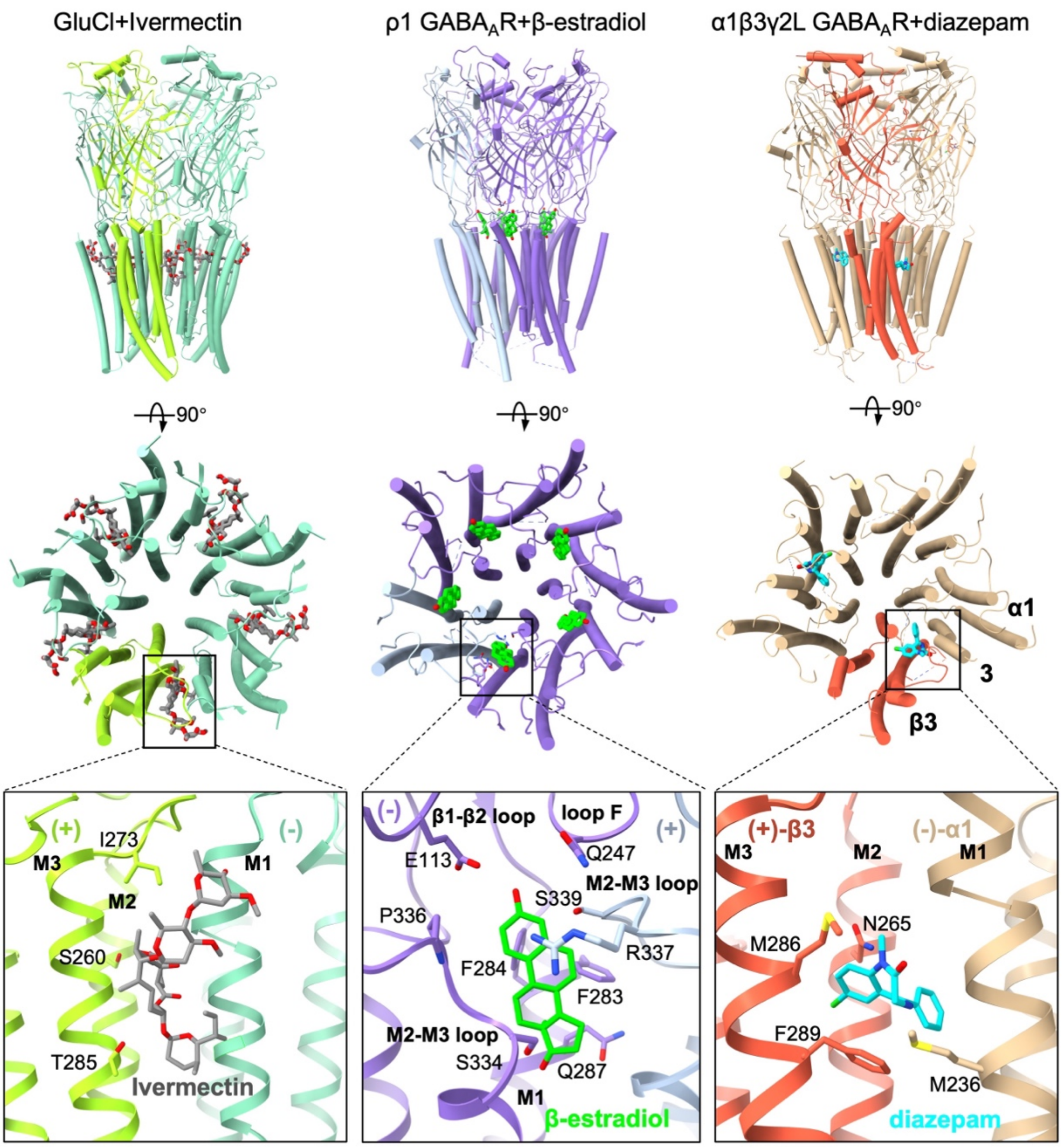

Supplementary Fig. S10. Examples of the structural basis for allosteric modulation through TMD sites in GluCl and GABA$_A$Rs.

The overall structures of GluCl/Ivermectin (left, PDB: 3RIF), ρ1 GABA$_A$R/β-estradiol (middle, PDB: 8RH7) and α1β3γ2L GABA$_A$R/diazepam (right, PDB: 6HUP) are shown. The top-down view of these structures highlights the arrangement of transmembrane helices (represented in tube mode) and the bound modulators (depicted as sticks). The binding sites are framed, and detailed view of receptor-modulator interactions are shown below in accordance.

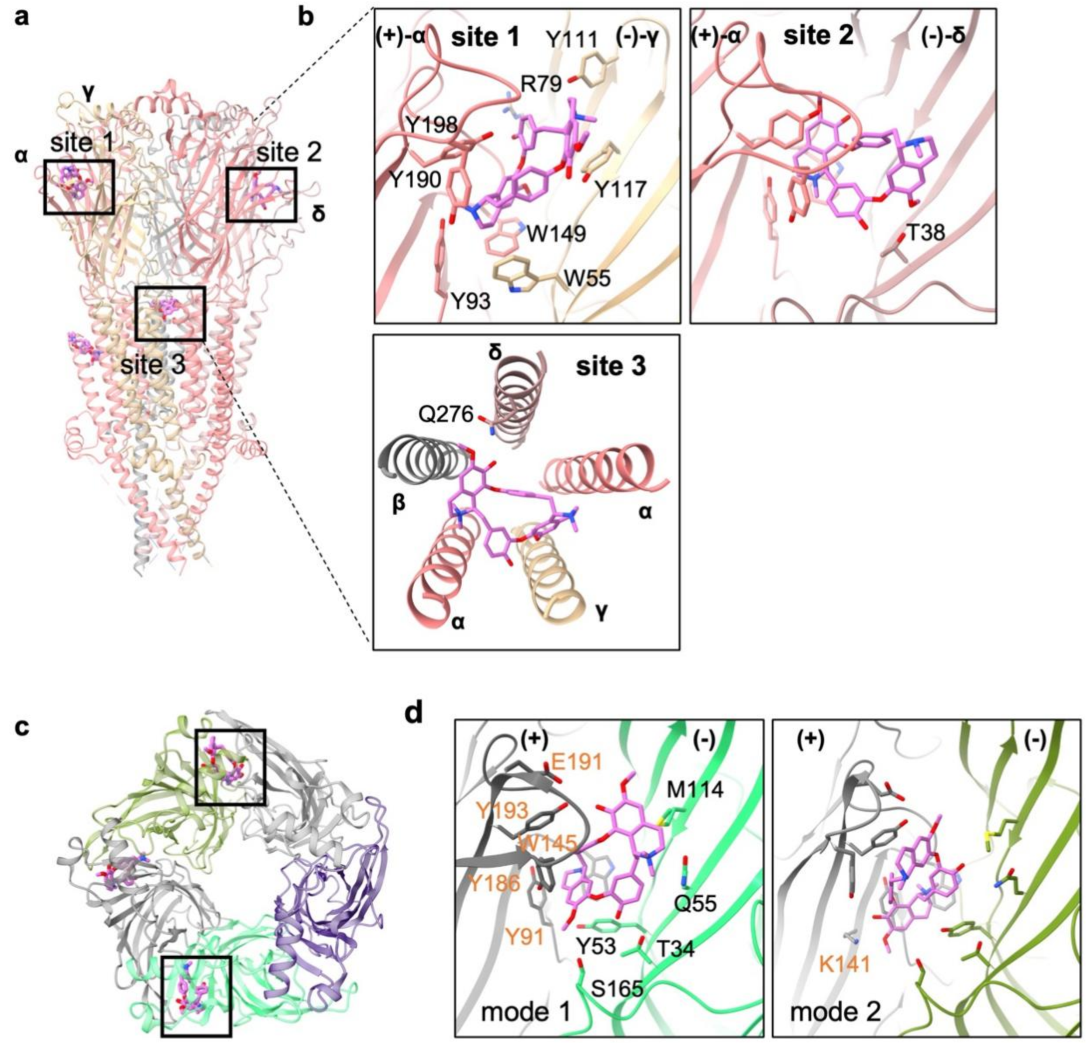

Supplementary Fig. S11. ***d*-TC binding sites in the α$_{(2)}$βγδ nAChR and AchBP.**

a, The overall structure of α$_{(2)}$βγδ nAChR/*d*-TC (PDB: 7SMS). **b,** The detailed structure information of *d*-TC binding site 1 (top, left), site 2 (top, right), site 3 (bottom). **c,** The overall structure of AchBP/*d*-TC (PDB: 2XYT). **d,** The detailed structure information of *d*-TC in binding mode 1 (left), mode 2 (right). *d*-TC is shown in orchid sticks. The principal subunit interface (+) and the complementary subunit interface (-) are colored differently.

	ZAC-Apo	ZAC-Zn	ZAC-TC	ZAC-TTFB	ZAC[A152]-Zn	ZAC-$Zn_{partial}$
**Data collection and processing**						
Magnification	105,000	105,000	105,000	130,000	105,000	130,000
Voltage (kV)	300	300	300	300	300	300
Electron exposure (e^-/Å^2)	50	50	50	50	50	50
Defocus range (µm)	-2.0	-2.0	-2.0	-2.0	-2.0	-2.0
Pixel size (Å)	0.932	0.932	0.932	0.932	0.832	0.932
Symmetry imposed	C1	C1	C1	C1	C5	C1
Final particle images (no.)	16,419	164,569	68,294	133,386	63,112	111,743
Map resolution (Å)*	3.35	2.62	2.97	2.54	2.63	2.85
**Refinement**						
Initial model used (PDB)	AlphaFold model	ZACN-Apo	ZACN-Apo	ZACN-Apo	ZACN-Apo	ZACN-Apo
Map sharpening *B* factor (Å^2)	69.5	89.9	71.4	80.4	94.3	83.9
Model composition						
non-hydrogen atoms	12,685	12,745	12,830	12,840	12,735	12,742
Protein residues	1,555	1,555	1,555	1,555	1,555	1,555
Ligands	BMA: 5; NAG:20;**	Zinc: 5; BMA: 10; NAG:20; **	TC9: 2; BMA: 10; NAG:20;**	Zinc: 5; TFB: 5 BMA: 10; NAG:20;**	Zinc: 5; BMA: 10; NAG:20;**	Zinc: 2; BMA: 10; NAG:20;**
B factor (Å^2)						
Protein	100.41	32.48	32.48	32.48	32.48	32.48
Ligand	118.11	57.22	66.89	58.01	56.30	56.57
R.m.s. deviations						
Bond lengths (Å)	0.003	0.007	0.006	0.006	0.004	0.006
Bond angles (°)	0.640	1.091	1.159	1.159	0.952	1.114
Validation						
MolProbity score	1.49	1.52	1.16	1.65	1.48	1.56
Clashscore	9.28	6.39	6.74	7.46	6.48	5.97
Poor rotamers (%)	0	0	0	0	0	0
Ramachandran plot						
Favored (%)	98.11	97.07	96.42	96.35	97.39	96.42
Allowed (%)	1.89	2.93	3.58	3.65	2.61	3.58
Outliers (%)	0.00	0.00	0.00	0.00	0.00	0.00
Deposited model (PDB id)	9LEV	9LET	9LEY	9LEZ	9LEU	9LEX
Deposited map (EMDB id)	63035	63033	63037	63038	63034	63036

Supplementary Table S1. Cryo-EM data collection and refinement statistics.

* Gold standard FSC with threshold of 0.143

** BMA: beta-D-mannopyranose ; NAG:2-acetamido-2-deoxy-beta-D-glucopyranose ; TC9: d-tubocurarine; TTFB:N-(4-(tert-butyl)thiazol-2-yl)-3-fluorobenzamide

hZAC - this study

*h*ZAC residue	Conserved	Not Conserved
Zinc-binding site		
Phe67	86 (86 x Phe)	2 (2 x Ser)
Arg88	60 (60 x Arg)	28 (19 x Lys, 9 x Gln)
Tyr176	88 (83 x Tyr, 5 x Phe)	0
Glu194	85 (85 x Glu)	3 (2 x Gln, 1 x Arg)
Disulphide Bond (M2-3 linker/C-terminal)		
Cys289	88 (88 x Cys)	0
Cys394	88 (88 x Cys)	0
Thr152 Network		
Leu126	88 (88 x Leu)	0
Arg131	30 (30 x Arg)	58 (51 x Gln, 7 x Lys)
Thr152	88 (88 x Thr)	0

hZAC - Lu *et al.*, 2025

*h*ZAC residue	Conserved	Not Conserved
Zinc-binding site 1		
Asp140	74 (74 x Asp, 4 x His)	14 (2 x Asn, 4 x Gly, 2 x Tyr, 2 x Phe)
Asp142	81 (81 x Asp)	7 (7 x Asn)
His145	80 (80 x His)	8 (3 x Gln, 2 x Leu, 3 x Arg)
Zinc-binding site 2		
His163	19 (19 x His)	69 (65 x Gln, 2 x Lys, 1 x Leu, 1 x Arg)
His168	84 (84 x His)	4 (4 x Arg)

olZAC structure - Jin *et al.*, 2024

*ol*ZAC residue	*h*ZAC residue	Conserved	Not Conserved
Zinc-binding candidates			
Asp103	Glu113	88 (77 x Asp,11xGlu)	1 (1 x Gly)
Glu148	Asn158	60 (60 x Asp)	29 (1 x Asn)
Glu160	Asn170	55 (48 x Asp, 7xHis)	34 (34 x Asn)
Glu204	Pro212	0	89 (85 x Pro, 4 x Leu)

Supplementary Table S2. Conservation of selected residues in ZAC across human ZAC and 88 non-human mammalian ZACs. The data is based on a amino acid sequence alignment of human ZAC and 88 non-human mammalian ZAC proteins. The 88 sequences were the remaining of the first 100 identified sequences in a NCBI Protein Blast search using the human ZAC sequence as query after removal of duplicate sequences, and the mammalian species and NCBI accession numbers for the 89 sequences are given below. Residues proposed to be involved in zinc-binding, in a disulfide-bond between two cysteines in the M2-3 linker and the C-terminal, and in the Thr152-associated network in human ZAC in this study (in black) are given together with residues proposed to be involved in zinc-binding to human ZAC (in blue) and to *ol*ZAC (in red) in two recent studies. The degree of conservation of each of the residues are for this study and Lu *et al.*, 2025 study given as the number of sequences where the human ZAC residue is “conserved” (incl. pseudo-conserved) and “not conserved” across the 88 non-human mammalian ZAC sequences and for the Jin *et al.*, 2024 study as the number of sequences where the olZAC residue is “conserved” (incl. pseudo-conserved) and “not conserved” across the 89 human and non-human mammalian ZAC sequences.

The species and NCBI accession numbers for the human ZAC and the 88 non-human mammalian ZAC proteins sequences: Human/*Homo sapiens* [KAI2585163.1], Bonono/*Pan paniscus* [XP_003813307.1], Gorilla/*Gorilla gorilla gorilla* [XP_004041081.3], Siamang/*Symphalangus syndactylus* [055098132.1], Silveri gibbon/*Hylobates moloch* [XP_032033685.2], Sumatran orangutang/*Pongo abelii* [XP_024090176.3*],* Northern white-cheeked gibbon/*Nomascus leucogenys* [XP_003279181.2], Green monkey/*Chlorocebus sabaeus* [XP_008009872.2], Tibetan macaque/*Macaca thibetana thibetana [*XP_050618330.1*],* Drill/*Mandrillus leucophaeus* [XP_011838800.1], Olive baboon/*Papio anubis* [XP_003913518.1], Gelada/*Theropithecus gelada* [XP_025219548.1], Crab-eating macaque/*Macaca fascicularis* [XP_005585096.3], Golden snub-nosed monkey/*Rhinopithecus roxellana* [XP_010385845.1], Sooty mangabey/*Cercocebus atys* [XP_011897785.1], Southern pig-tailed macaque/*Macaca nemestrina* [*XP_011718230.1*], Angola colobus/*Colobus angolensis* [XP_011804714.1], Ugandan red colobus/*Piliocolobus tephrosceles* [XP_023067530.1], Rhesus macaque/*Macaca mulatta* [XP_014975902.2], Black-capped squirrel monkey/*Saimiri boliviensis* [XP_003931764.1], Tufted capuchin/*Sapajus apella* [XP_032097506.1], Philippine tarsier/*Carlito syrichta* [XP_008058784.1], Cotton-top tamarin/*Saguinus oedipus* [KAK2112095.1], Nancy Ma's night monkey/*Aotus nancymaae* [XP_012309341.3], Common marmoset/*Callithrix jacchus* [XP_008996022.3]), Aye-aye/*Daubentonia madagascariensis* [KAL2764619.1], Coquerel's sifaka/*Propithecus coquereli* [XP_012517792.1], Greater horseshoe bat/*Rhinolophus ferrumequinum* [XP_032946523.1], Northern greater galago/*Otolemur garnettii* [XP_003786159.1], Brown bear/*Ursus arctos* [XP_026339886.3], Ring-tailed lemur/*Lemur catta* [XP_045382996.1], Sunda slow loris/*Nycticebus coucang* [XP_053426761.1], Coppery titi monkey/*Plecturocebus cupreus* [KAL0594676.1], Cat/*Felis catus* [XP_011287686.2], Lion/*Pantera leo* [XP_042771513.1], Canada lynx*/Lynx canadensis* [XP_030150948.1], American black bear/*Ursus americanus* [XP_045670155.1], Bobcat/*Lynx rufus* [XP_046937303.1], Tiger/*Panthera tigris* [*XP_007091340.1*], Leopard*/Panthera pardus* [*XP_019321690.2],* Jaguar/*Panthera onca* [XP_060501313.1], Cheetah/*Acinonyx jubatus* [XP_053068366.1], Red-fronted lemur/*Eulemur rufifrons* [XP_069338985.1], Northern sea otter/*Enhydra lutris kenyoni* [*XP_022379206.1*], Gray mouse lemur/*Microcebus murinus* [XP_012634152.1], Red fox/*Vulpes vulpes* [XP_025855589.1], Fishing cat/*Prionailurus viverrinus* [XP_047689851.1], Leopard cat/*Prionailurus bengalensis* [XP_043441569.1], Dog/*Canis lupus familiaris* [NP_001010955.1], Stoat/*Mustela erminea* [XP_032175686.1], Clouded leopard/*Neofelis nebulosa* [XP_058558778.1], Ferret/*Mustela putorius furo* [XP_004748917.1], Common raccoon dog/*Nyctereutes procyonoides* [CAD7668287.1], European mink/*Mustela lutreola* [XP_059006337.1], Greater mouse-eared bat/*Myotis myotis* [XP_036195361.1], Jaguarundi/*Puma yagouaroundi* [XP_040312416.1], European badger/*Meles meles* [XP_045840777.1], Polar bear/*Ursus maritimus* [XP_008692015.2], American Eur/*Neogale vison* [XP_044103326.1], David's myotis/*Myotis davidii* [ELK35763.1], Striped hyena/*Hyaena hyaena* [XP_039082202.1], Greater sac-winged bat/*Saccopteryx bilineata* [XP_066093636.1], Arctic fox/*Vulpes lagopus* [XP_041582791.1], Velvety free-tailed bat/*Molossus molossus* [KAF6417674.1], Brandt's myotis/*Myotis brandtii* [XP_005884292.2], Giant panda/*Ailuropoda melanoleuca* [XP_002919937.3], Spotted hyena/*Crocuta crocuta* [KAF0887047.1], Northern elephant seal/*Mirounga angustirostris [*XP_045743962.2*],* Horse/*Equus caballus* [*XP_014593025.2*], Lesser white-lined bat/*Saccopteryx leptura* [XP_066238006.1], Meerkat/*Suricata suricatta* [XP_029783277.1], Plains zebra/*Equus quagga* [XP_046532299.1], Mongolian wild horse/*Equus przewalskii* [XP_008540513.2], Geoffroy's cat/*Leopardus geoffroyi* [XP_045345643.1], Spanish mole/*Talpa occidentalis* [XP_037349236.1], naked mole-rat/*Heterocephalus glaber* [EHA99417.1], North Atlantic right whale/*Eubalaena glacialis* [XP_061030670.1], Northern treeshrew/*Tupaia chinensis belangeri [*ELW68295.1], Southern elephant seal/*Mirounga leonina* [XP_034882286.1], Yuma myotis/*Myotis yumanensis* [XP_070251517.1], North American river otter/*Lontra canadensis* [XP_032726459.1], Weddell seal/*Leptonychotes weddellii* [XP_006728112.1], Damaraland mole-rat/*Fukomys damarensis* [XP_033623316.1], Harbor seal/*Phoca vitulina* [XP_032285733.1], Northern bat/*Cnephaeus nilssonii* [KAK1330807.1], Thirteen-lined ground

squirrel/*Ictidomys tridecemlineatus* [XP_040124570.1], Steller sea lion/*Eumetopias jubatus* [XP_027968121.1], Yellow-bellied marmot/*Marmota* flaviventer [XP_027811455.1], South-central black rhinoceros/*Diceros bicornis minor* [XP_058417678.1].

	EC_{50} (mM)	pEC_{50} ± S.E.M.	n	Functional characteristics
**WT**	0.269	[3.57 ± 0.03]	29	-
**F67A**	>10	[<2.0]	25	Inactive [a]
**F67H**	>10	[<2.0]	10	Inactive [a]
**F67W**	0.095	[4.02 ± 0.09]	8	Left-shifted CRC compared to WT
**R88A**	>10	[<2.0]	19	Inactive [a]
**R88L**	>10	[<2.0]	10	Inactive [a]
**R88K**	0.138	[3.86 ± 0.10]	12	Left-shifted CRC compared to WT
**Y176A**	>10	[<2.0]	17	Inactive [a]
**Y176H**	>10	[<2.0]	10	Inactive [a]
**Y176W**	0.03-0.1 [b]		10	Left-shifted CRC and increased spontaneous activity compared to WT
**E194A**	n.d.	n.d.	31	Active but CRC is not determinable [c]
**E194L**[a]	>10	[<2.0]	12	Inactive [a]
**E194Q**	0.274	[3.56 ± 0.06]	8	WT-like CRC

Supplementary Table S3. Agonist properties displayed by Zn^{2+} at wild-type (WT) ZAC and ZAC mutants comprising mutations of the Zn^{2} site-forming residues Phe67, Arg88, Tyr176 and Glu194 expressed in *Xenopus* oocytes in TEVC electrophysiological recordings. EC_{50} (in mM) and pEC_{50} ± S.E.M. values are given, and the number of experiments for the data (i.e., number of recorded oocytes, n) are indicated. CRC, concentration-response relationship. n.d., not determinable.

[a] Zn^{2+} did not evoke significant current responses through these mutants at concentrations up to 10 mM.

[b] The complex CRC exhibited by Zn^{2+} at Y176W made precise determinations of EC_{50} values difficult, and thus an approximate EC_{50} concentration range is given for this mutant.

[c] Zn^{2+} evoked very minute but significant current responses through E194A in the same concentration range as through WT ZAC (0.03-10 mM).

	EC_{50} (mM)	pEC_{50} ± S.E.M.	n
**WT**	0.653	[3.19 ± 0.03]	8
**T152A**	~0.3-1.0 [a]	[~ 3.5-3.0] [a]	5
**T152D**	~0.3-1.0 [a]	[~ 3.5-3.0] [a]	6
**L126P**	0.413	[3.38 ± 0.04]	7
**L126A**	0.892	[3.05 ± 0.03]	9
**R131A**	0.355	[3.45 ± 0.10]	6
**R131Q**	0.672	[3.17 ± 0.06]	5

Supplementary Table S4. Agonist properties displayed by Zn^{2+} at wild-type (WT) ZAC and ZAC mutants comprising mutations of the residues Thr152, Leu126 and R131 expressed in *Xenopus* oocytes in TEVC electrophysiological recordings. EC_{50} (in mM) and pEC_{50} ± S.E.M. values are given, and the number of experiments for the data (i.e., number of recorded oocytes, n) are indicated

[a] Because of the minute current amplitudes evoked by Zn^{2+} through this mutant, it was not always possible to determine the agonist EC_{50} reliably based on the concentration-response curves, and thus intervals for the EC_{50} and pEC_{50} values are given.

	Zn^{2+}		TTFB	
	EC$_{50}$ (mM)	pEC$_{50}$ ± S.E.M.$^{(n)}$	IC$_{50}$ (μM)	pIC$_{50}$ ± S.E.M. $^{(n)}$
**WT**	0.342	[3.47 ± 0.04] $^{(13)}$	4.8	[5.32 ± 0.06]$^{(9)}$
**S277A**	0.942	[3.03 ± 0.05] $^{(8)}$	5.7	[5.24 ± 0.04] $^{(6)}$
**S278A**	0.533	[3.27 ± 0.06] $^{(7)}$	9.9	[5.00 ± 0.05] $^{(6)}$
**S277G/S278G/Q281G**	0.395	[3.40 ± 0.01] $^{(5)}$	13	[4.89 ± 0.07] $^{(10)}$

Supplementary Table S5. Agonist properties displayed by Zn^{2+} and antagonist properties displayed by TTFB at wild-type (WT) ZAC and ZAC mutants comprising mutations of residues in the putative TTFB binding site expressed in *Xenopus* oocytes determined by TEVC electrophysiology. EC_{50} and pEC_{50} ± S.E.M. values for Zn^{2+} and the fitted IC_{50} and pIC_{50} ± S.E.M. values for TTFB in its inhibition of the Zn^{2+} (1 mM)-evoked responses through the receptors are given with the number of experiments for the data (i.e., number of recorded oocytes, n) in superscript. The TTFB data for WT and mutant ZACs were determined in parallel at the same oocyte batches and are given in Supplementary Fig. S9.

[a] The L273A, V274A and L275A mutants were also constructed to investigate the putative TTFB site: However, injection of cRNAs for these mutants resulted consistently in oocytes of insufficient quality to enable TEVC recordings from them.

	Zn^{2+}		*d*-TC	
	EC$_{50}$ (mM)	pEC$_{50}$ ± S.E.M.$^{(n)}$	IC$_{50}$ (μM)	pIC$_{50}$ ± S.E.M. $^{(n)}$
	***d*-TC Site 1 (ECD)**[a]			
**WT**	0.342	[3.47 ± 0.04] $^{(13)}$	3.6	[5.44 ± 0.06] $^{(14)}$
**R65A**	0.389	[3.41 ± 0.08] $^{(8)}$	3.4	[5.47 ± 0.04] $^{(10)}$
**R65L**	0.591	[3.23 ± 0.06] $^{(6)}$	3.0	[5.52 ± 0.02] $^{(6)}$
**R65W**	0.662	[3.18 ± 0.07] $^{(5)}$	2.8	[5.55 ± 0.09] $^{(6)}$
**N148A**	0.761	[3.12 ± 0.09] $^{(6)}$	4.4	[5.35 ± 0.08] $^{(9)}$
**N148L**	0.548	[3.26 ± 0.06] $^{(5)}$	3.1	[5.51 ± 0.08] $^{(6)}$
**N148W**	0.581	[3.24 ± 0.06] $^{(5)}$	2.1	[5.67 ± 0.03] $^{(6)}$
**L178A**	0.292	[3.53 ± 0.09] $^{(6)}$	3.9	[5.40 ± 0.03] $^{(7)}$
**L178K**	0.211	[3.68 ± 0.11] $^{(5)}$	3.6	[5.44 ± 0.09] $^{(6)}$
**Q214A**	0.848	[3.07 ± 0.03] $^{(5)}$	3.2	[5.49 ± 0.07] $^{(7)}$
**Q214K**	0.406	[3.39 ± 0.04] $^{(5)}$	2.1	[5.68 ± 0.06] $^{(6)}$
**L216A**	0.568	[3.25 ± 0.03] $^{(6)}$	4.7	[5.33 ± 0.04] $^{(7)}$
**R65L/N148L**	0.669	[3.17 ± 0.06] $^{(6)}$	3.6	[5.44 ± 0.09] $^{(6)}$
**R65L/N148L/Q214K**	0.421	[3.38 ± 0.06] $^{(9)}$	4.5	[5.34 ± 0.08] $^{(10)}$
	***d*-TC Site 2 (TMD)**[b]			
**WT**	0.302	[3.52 ± 0.04] $^{(12)}$	3.0	[5.52 ± 0.06] $^{(17)}$
**S277A**	0.942	[3.03 ± 0.05] $^{(8)}$	4.2	[5.38 ± 0.07] $^{(6)}$
**S278A**	0.533	[3.27 ± 0.06] $^{(7)}$	2.7	[5.57 ± 0.07] $^{(7)}$
**Q281G**	0.521	[3.28 ± 0.04] $^{(7)}$	3.9	[5.41 ± 0.01] $^{(6)}$
**Q281L**	0.843	[3.07 ± 0.02] $^{(8)}$	4.0	[5.39 ± 0.03] $^{(6)}$
**Q281K**	0.782	[3.11 ± 0.03] $^{(5)}$	7.6	[5.12 ± 0.02] $^{(7)}$
**S277G/S278G/Q281G**	0.395	[3.40 ± 0.01] $^{(5)}$	3.1	[5.51 ± 0.05] $^{(9)}$
	***d*-TC Sites 1+2 (ECD+TMD)**			
**R65L/N148LQ214K/S277G/S278G/Q281G**	0.237	[3.63 ± 0.05] $^{(6)}$	3.2	[5.50 ± 0.05] $^{(7)}$

Supplementary Table S6. Agonist properties displayed by Zn^{2+} and antagonist properties displayed at *d*-tubocurarine (*d*-TC) at wild-type (WT) ZAC and ZAC mutants comprising mutations of residues in the putative *d*-TC binding sites 1 and/or 2 expressed in *Xenopus* oocytes determined by TEVC electrophysiology. EC$_{50}$ and pEC$_{50}$ ± S.E.M. values for Zn^{2+} as agonist and the fitted IC$_{50}$ and pIC$_{50}$ ± S.E.M. values for *d*-TC in its inhibition of the Zn^{2+} (1 mM)-evoked response through the receptors are given with the number of experiments for the data (i.e., number of recorded oocytes, n) in superscript. The specific WT ZAC data given for comparison were determined in parallel with at the same oocyte batches as the site 1 and site 2 mutants. Data for the *d*-TC experiments are given in Supplementary Figs. S5-6.

[a] The following mutants also constructed to investigate *d*-TC site 1 were found to be inactive (non-responsive to Zn^{2+} at concentrations up to 10 mM): Q214L, Q214W, L216R. Moreover, injection of oocytes with L123A, L123K or V217A ZAC cDNA consistently resulted in oocytes of insufficient quality to enable TEVC recordings from them.

[b] The following additional mutants constructed to investigate *d*-TC site 2 were found to be inactive (non-responsive to Zn^{2+} at concentrations up to 10 mM): S277L, S277W, S278L, S278W and S277V/S278V/Q281V. The V274A mutant was also constructed to investigate *d*-TC site 2: However, injection of cRNA for this mutant resulted consistently in oocytes of insufficient quality to enable TEVC recordings from them.
